# A Study of the Micellar Formation of *N*-Alkyl Betaine Ethyl Ester Chlorides Based on the Physicochemical Properties of Their Aqueous Solutions

**DOI:** 10.3390/molecules29081844

**Published:** 2024-04-18

**Authors:** Monika Geppert-Rybczyńska, Anna Mrozek-Wilczkiewicz, Patrycja Rawicka, Piotr Bartczak

**Affiliations:** 1 Institute of Chemistry, University of Silesia, Szkolna 9, 40-006 Katowice, Poland; 2 August Chełkowski Institute of Physics, University of Silesia, 75 Pułku Piechoty 1, 41-500 Chorzów, Poland; anna.mrozek-wilczkiewicz@us.edu.pl (A.M.-W.); patrycja.rawicka@us.edu.pl (P.R.); 3 Department of Systems Biology and Engineering, Silesian University of Technology, Akademicka 16, 44-100 Gliwice, Poland; 4 Centre for Materials and Drug Discovery, Institute of Chemistry, University of Silesia, Szkolna 9, 40-006 Katowice, Poland; piotr.bartczak@us.edu.pl

**Keywords:** betaine surfactants, viscosity, speed of sound, surface tension, dynamic light scattering, intermolecular interactions, critical micelle concentration

## Abstract

In this study, a series of four surface-active compounds—*N*-alkyl betaine ethyl ester chlorides, C*_n_*BetC_2_Cl—were synthesized and characterized in aqueous solutions. As with other alkyl betaines, these amphiphiles can be practically used, for example, as co-surfactants and/or solubility enhancers acting according to hydrotropic or micellar mechanisms, depending on the alkyl chain length in the amine. We focused on the representatives of the medium alkyl chain length (C_6_–C_12_) to find the dependence between the alkyl chain length in *N*-alkyl betaine ethyl ester chlorides and the surface, volumetric, acoustic, and viscometric properties of their solutions. Ethyl esters, the derivatives of amino acids, were chosen to increase functionality and take advantage of possible hydrolysis in solutions at higher pH, which is also a key parameter in biodegradability. The micellization parameters were calculated based on the physicochemical characteristics. We focused our interest on the ester with a dodecyl substituent since we can compare and discuss its properties with some other C_12_ representatives that are available in literature. Surprisingly, its micellization characteristic is almost temperature-independent in the investigated temperature range, *t* = (15–45) °C. Particularly interesting are the results of dynamic light scattering (DLS), which show that the changes in physicochemical parameters of the C_12_ homolog around the CMC are caused by the two types of micelles of different sizes present in solutions.

## 1. Introduction

Surface-active compounds (surfactants) affect the surface tension of solvents. The most common is water, with its unique properties, such as the relatively high surface tension, which can be decreased by adding surfactant. The molecules of surfactants have an amphiphilic character resulting from hydrophobic (lipophilic) and hydrophilic polar moieties in their structure. Surfactants based on compounds occurring in natural sources are regarded as more environmentally friendly due to the expected facilitated degradation process. It can be attainable because of the high levels of carbon dioxide formation and loss of dissolved organic carbon, indicating total biological breakdown without forming hard residues [1,2,3]. In surfactants based on amino acids, the natural polar head group is substituted to the hydrophobic tail: an ester or an amide bond [4]. Another possibility to ensure the amphiphilic character, such as in *N*-alkyl betaines, is substituting the methyl group with an alkyl chain (hydrophobic tail) directly at the nitrogen atom of the amine group.

*N*-alkyl betaines, including those with some functional groups in the alkyl moiety, are an interesting example of surfactants. They are the subject of many investigations, including the size and shape of the micelles in solutions and their antimicrobial activity, since they can be obtained from natural resources and are potentially biodegradable [2,3,5,6,7,8,9,10,11,12]. Numerous representatives of alkyl betaines have been applied in cosmetics as antistatic agents, hair conditioning agents, skin conditioning agents, surfactant cleansing agents, surfactant foam boosters, and viscosity-increasing agents (aqueous), or in surfactant/polymer flooding systems [13,14,15]. However, alkyl betaines’ (amino acid) transformation into esters of variable length of alkyl chain in the carboxyl group can modify their properties and open a new range of possible applications [16,17,18,19,20].

The betaine esters seem more advantageous than the alkyl betaines; moreover, they can be obtained directly from their esters. An ester group is more beneficial than a carboxyl, having better reactivity since it provides better solubility of less polar compounds [12]. However, the alkyl betaine esters can hydrolyze at a higher pH [4,19,21], which should be considered in their surfactant applications.

Chosen betaine esters, or betaine ester halides (usually chlorides or bromides), especially with alkyl chain (*R* = C*_n_*H_2*n*+1_) in the ester group (R-OOCCH_2_N^+^(CH_3_)_3_X^−^) have also been the subject of numerous investigations [16,17,19,20]. For some betaine derivatives, alkaline hydrolysis can occur, limiting their use where surfactant degradation is desirable [2,19]. The micellization of alkyl betaines and some ester bromides (C_8_–C_18_) in water has been studied so far using different techniques, and the critical micelle concentration (CMC) at 25 °C has also been reported [7,17,20,22].

**Scheme 1:**
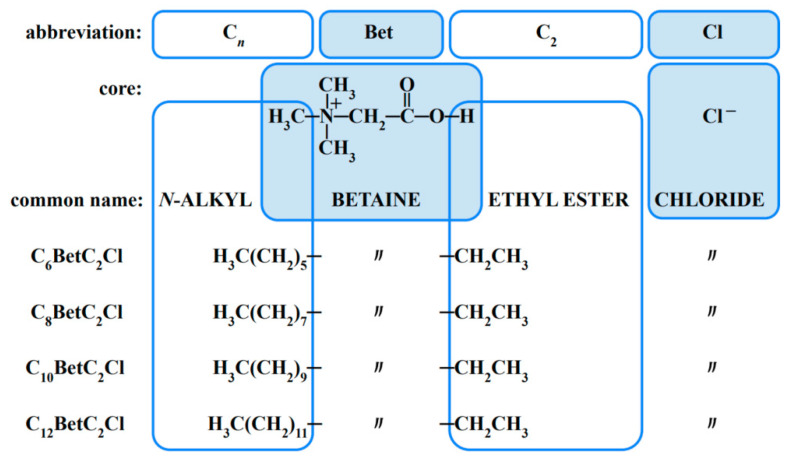
Chemical structures of the *N*-(2-ethoxy-2-oxoethyl)-*N*,*N*-dimethyl-alkyl-1-aminium chlorides, C*_n_*BetC_2_Cl (*n* = 6, 8, 10, 12) investigated in aqueous solutions.

In the first part of this study, there are presented complex investigations of the physicochemical properties—including volumetric, acoustic, tensiometric, viscometric, and optical (by use of dynamic light scattering (DLS)) properties of the aqueous solutions of C*_n_*BetC_2_Cl at 25 °C—of four *N*-(2-ethoxy-2-oxoethyl)-*N*,*N*-dimethyl-alkyl-1-amminium chlorides C*_n_*BetC*_2_*Cl (*n* = 6, 8, 10, 12) (Scheme 1), together with a description of the synthesis of these amphiphiles.

Since C*_n_*BetC_2_Cl compounds have melting points below 100 °C, they can also be regarded as ionic liquids, or surface active ionic liquids, SAILs, with all the properties of this class of compounds [23,24]. Moreover, in these salts, pH changes do not affect the electrical positive charge on the molecule, so they share most of the properties and applications of cationic surfactants. Based on the expected behavior of amphiphiles, one may presume that the change in the length of the hydrocarbon chain of surfactant (*N*-(2-ethoxy-2-oxoethyl)-*N*,*N*-dimethyl-alkyl-1-aminium chlorides) influences not only micelle size and aggregation in aqueous solutions but also their physicochemical properties required for their different applications.

In the second part, the effect of temperature on the properties mentioned above for C_12_BetC_2_Cl aqueous solutions is reported at *t* = (15–45) °C. C_12_BetC_2_Cl is our starting point since it is one of many possible derivatives of the very popular zwitterionic lauryl betaine (*N*-dodecyl betaine) [3,7], a mild equivalent of sodium lauryl sulfate (sodium dodecyl sulfate, SLS), which is used in natural and synthetic cosmetics [13,25]. Lauryl betaine is also effective in antimicrobial activity [9] and oil recovery due to high foaming [25]. The C_12_ length of the alkyl chain is also essential for the following reasons. On one hand, the homologs C16 and higher often have better properties as surfactants and a lower CMC. On the other hand, their water solubility decreases, and their activity against some bacteria is sometimes slightly lower than C_12_ [9]. The sizes of micelles presumably cause this effect—which is more significant for longer chain homologs—affecting their diffusibility and permeation abilities on the microbial cell wall [9]. Eventually, surfactants with a dodecyl moiety are often discussed regarding the influence of its composition on micellar properties [26].

The chosen properties of one of the compounds presented in this report have already been presented for C_10_BetC_2_Cl [22], where the authors focused on calorimetry and dielectric spectroscopy investigations of the hydration process of a set of some quaternary ammonium chlorides in the broader temperature range. When necessary, we compare our results with those obtained by Medos et al. (investigations of the hydration process of a set of some quaternary ammonium chlorides [22]) and provide a detailed discussion. To complete the picture of the micellization process by giving an orientation about the size distribution profile of micelles in solution in C_12_BetC_2_Cl and C_10_BetC_2_Cl, we analyzed the results of dynamic light scattering (DLS) measurements. All results obtained will be discussed based on a comparison with the equivalent properties of solutions of ionic surfactants, including alkyl betaines and their derivatives, reported in the literature.

Based on the results described in this text and cited literature, we plan to investigate the potential applications of *N*-alkyl betaine ester salts as biostatics [27,28] and in mixed micellization with some anti-inflammatory drugs [29].

## 2. Results and Discussion

### 2.1. Density and Speed of Sound Measurements

**Figure 1:**
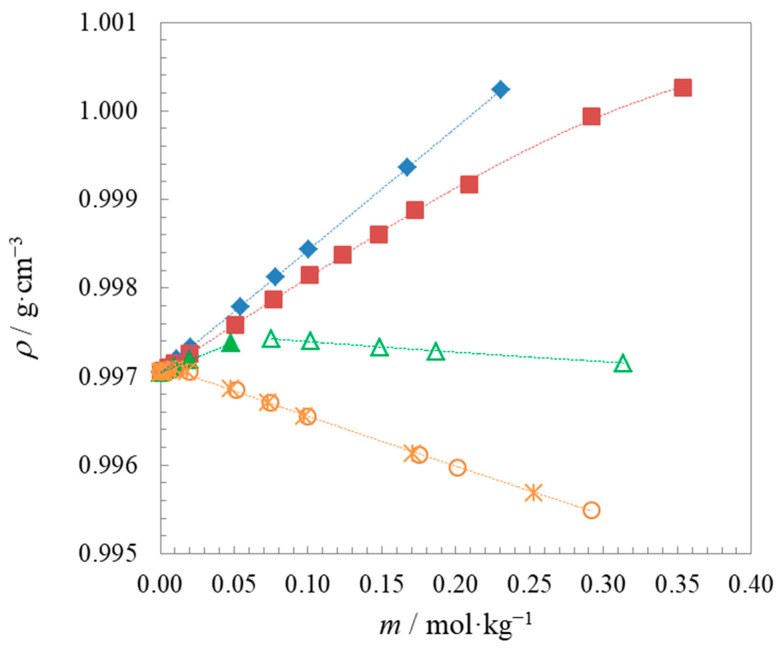
Density of aqueous solutions of C*_n_*BetC_2_Cl at *t* = 25 °C; experimental points: (♦) *n* = 6, (■) *n* = 8, (▲, Δ) *n* = 10, (●, ○) *n* = 12 (second series), (∗) *n* = 12 (first series); filled symbols—before breakdown (at CMC), empty symbols—after breakdown (at CMC); lines—1st or 2nd order polynomials (see Table S3 in the Supplementary Materials).

**Figure 2:**
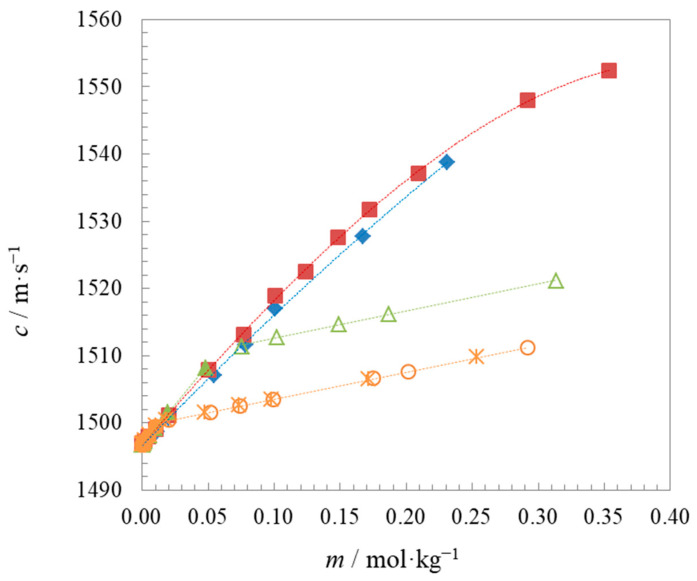
Speed of sound in aqueous solutions of C*_n_*BetC_2_Cl at *t* = 25 °C; experimental points: (♦) *n* = 6, (■) *n* = 8, (▲, Δ) *n* = 10, (●, ○) *n* = 12 (second series), (∗) *n* = 12 (first series); filled symbols—before breakdown (at CMC), empty symbols—after breakdown (at CMC); lines—1st order polynomials (see Table S3 in the Supplementary Materials).

The density, *ρ*, and speed of sound, *c*, in aqueous solutions of *N*-alkyl betaine ethyl ester chlorides (*n* = 6–12) were at first measured at 25 °C, and then the solutions of the homolog C_12_BetC_2_Cl (prepared from the second stock solution) were also measured in the temperature range *t* = (15–45) °C, with a step of 10 °C. The results are listed in Figure 1 and Figure 2, Table S2, Figures S21 and S22 in the Supplementary Materials.

The density of one of the compounds, C_10_BetC_2_Cl, has previously been reported in the literature [22]. The measurements were performed in the temperature range *t* = (5–55) °C, with a step of 10 °C, in addition to calorimetry and dielectric relaxation spectroscopy applied to a series of aqueous solutions of functionalized quaternary ammonium chlorides. The concentration range presented by Medos et al. [22] was very wide (until *m* ≈ 1.22 mol∙kg^−1^), with only a few concentration points at a lower value of *m*. The comparison of density obtained in this study and the literature for values at 25 °C is presented in Figure S23 in the Supplementary Materials. The agreement between the two data sets is good at lower concentrations, with the breakdown of the *ρ*(*m*) curve placed nearly at the same value of *m*. In the paper by Medos et al. [22], only two points were present before this breakdown; thus, it was impossible to calculate the critical micelle concentration, CMC, from the density.

The density and speed of sound with concentration change depending on the length of the alkyl chain. Both can change monotonically or with a break on the curve caused by the aggregation processes in the solution with increasing *m* value. The polynomials of the form $y=\sum_{i=0}^{n=2}y_{i}\cdot m^{i}$ are fitted to *ρ*(*m*) and *c*(*m*). In order to find the CMC, the dependence of density and speed of sound on concentration were described separately for *m* before and after the CMC. The CMCs were then determined analytically as cross points of these polynomials. The separate regions were distinct only for C_10_BetC_2_Cl and C_12_BetC_2_Cl. For C_6_BetC_2_Cl and C_8_BetC_2_Cl, *ρ*(*m*) and *c*(*m*) dependences were described satisfactorily with one equation only in the investigated concentration range when the mean deviations from the regression lines were close to the uncertainty of density or speed of sound measurements (see last column in Table S3 in the Supplementary Materials). The coefficients of all dependencies are collected in Table S3 in the Supplementary Materials. They were also used in CMC calculations based on the density and speed of sound presented in Table 1.

Figure 1 shows that the density of C_10_ and C_12_ solutions after the CMC decreases with the concentration of electrolytes, which is not intuitive. The strong influence of the hydrophobic part of the amphiphilic solute on water and the increase in the distance between the solute and water molecules in the solution presumably causes it. At the same time, in Figure 2, the change of slope of *c*(*m*) curves is observed, indicating the increasing rigidity of the system and less effective ultrasound propagation in solution.

### 2.2. Surface Properties

**Figure 3:**
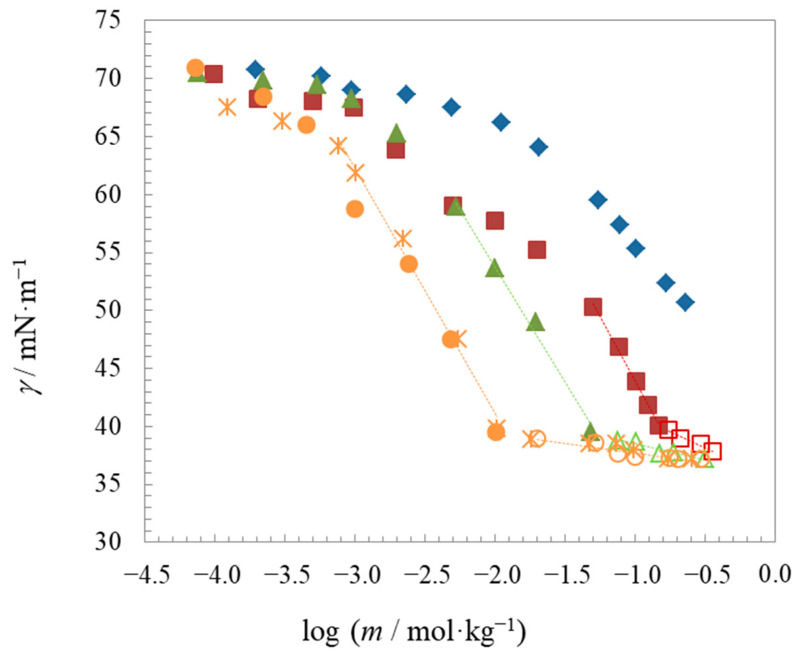
Surface tension of aqueous solutions of C*_n_*BetC_2_Cl at *t* = 25 °C; experimental points: (♦) *n* = 6, (■, □) *n* = 8, (▲, Δ) *n* = 10, (●, ○) *n* = 12 (second series), (∗) *n* = 12 (first series); filled symbols—before breakdown (at CMC), empty symbols—after breakdown (at CMC); lines—1st order polynomials (see Table S3 in the Supplementary Materials).

The surface tension values for aqueous solutions of C*_n_*BetC_2_Cl are collected in Table S3 and Figure 3. CMCs were calculated from the break point of the surface tension and the logarithm of the concentration dependence (*γ*(log*m*)) (Table 1). To determine the CMC, we took into account all points from the range where almost no variation of surface tension with log*m* is observed (at higher concentrations) and only those points that are close to the linear course (at lower concentrations). In Table 1 the surface tension value at the CMC (*γ*_CMC_) is listed, which means the effectiveness of surface tension reduction, and the surface excess, were calculated from the Gibbs adsorption isotherm equation:

(1) $$\Gamma_{max}=-\frac{1}{i\cdot 2.303RT}\cdot {\left(\frac{d\gamma }{d\;log\;log\;m\;}\right)}_{T,p}$$

where *γ* is the surface tension (mN∙m^−1^), *Γ* is the Gibbs excess surface concentration (mol∙m^−2^), *R* is the gas constant (8.314 J∙mol^−1^∙K^−1^), *T* is the absolute temperature (K), *m* is the molar surfactant concentration (mol∙kg^−1^) (assuming the activity coefficient is unity), and (d*γ*/dlog*m*) is the slope below the CMC in the *γ*(log*m*) plot; the value of *i* depends on the surfactant type: here it is taken as 2, as it is for 1:1 electrolytes and for ionic surfactants (for amino acids, *i* is equal to 1). Since the dependencies of the plots below, but close to the CMC, are linear (Figure 3 and Figure S24 in the Supplementary Material), the maximum surface excess concentration has been reached, *Γ*_max_ (Table 1).

In Table 1 there is also listed *A*_min_ (m^2^), the minimum area/molecule at the interface of water and air obtained from the saturation adsorption (*Γ*_max_), using the following equation:

(2) $$A_{min}=\frac{1}{N\cdot \Gamma_{max}}$$

where *N* is Avogadro’s number (6.022·10^23^ mol^−1^).

Finally, in Table 1, *p*C_20_—the negative log of the bulk surfactant concentration required to reduce the surface tension of the solvent by 20 mN∙m^−1^, i.e., the index of the efficiency of surface adsorption, ref. [30]—is presented.

The standard free energy of micellization (Δ*G_m_*) and adsorption for surfactants under study ∆*G*_ads_ in aqueous solution can be calculated using the following equation:

(3) $$∆G_{m}=RT\;ln\;ln\;x_{CMC}$$

where *x*_CMC_—is a molar fraction of surfactant at the CMC.

The knowledge of Δ*G_m_*, *Γ_max_* was used for the calculations of the free energy of adsorption, ∆*G_ads_*:

(4) $$∆G_{ads}=∆G_{m}-\frac{\Pi_{CMC}}{\Gamma_{max}}$$

where $\Pi_{CMC}=\gamma_{water}-\gamma_{CMC}$ is the surface pressure at the saturated air/solution interface.

Surface properties: Gibbs surface excess, critical micelle concentration (CMC) and related quantities characterize the surface activity of *N*-alkyl betaine ethyl ester chlorides and the micellization process.

Critical micelle concentration, CMC, of amphiphiles found in this study, especially for C_10_BetC_2_Cl and C_12_BetC_2_Cl, in aqueous solution calculated from the surface tension at *t* = 25 °C is at the *m* range characteristic for typical ionic surfactants with the decyl or dodecyl moiety [4,17,26,31], such as for dodecyltrimethylammonium bromide (15.3 ± 0.1 mmol∙kg^−3^) [32], or at least about one order higher than for *N*-alkyl betaines (170–1.8 mmol∙dm^−3^) [33,34], or one-third higher than for the equivalent surfactant sodium dodecyl sulfate, SDS (8 mmol∙dm^−3^) [35]. For dodecyl betaine, C_12_Bet, the CMC at *t* = 10 °C is 2 mmol∙dm^−3^ and increases with the temperature until it reaches 2.8 mmol∙dm^−3^ at *t* = 57 °C, which is not so clear for amphiphiles [36]. At the same time, CMC values C*_n_*BetC_2_Cl calculated from the density and speed of sound are close to the value found from (d*γ*/dlog*m*) dependence.

Also, the temperature dependence of the CMC for C_12_BetC_2_Cl is very weak, which may be more significant than the influence of the number of methylene groups in the alkyl chain. Generally, for *N*-alkyl betaine ethyl ester chlorides, the CMC is decreased two to three times by adding two methylene groups to the alkyl chain. In contrast, for typical ionic surfactants, the CMC is decreased by one order under the same condition [33,37]. The CMC calculated by applying a two-step micellization model to the isothermal titration calorimetry for C_10_BetC_2_Cl at *t* = 25 °C was (0.053 ± 0.001) mol∙L^−1^, as was reported by Medos et al. [22]. Such a value agrees with the results shown here, especially those calculated from concentration dependence of surface tension. The temperature dependence of the CMC of C_10_BetC_2_Cl is also very weak (from (0.068 ± 0.002) mol∙L^−1^ at *t* = 5 °C to (0.055 ± 0.003) mol∙L^−1^ at *t* = 55 °C), as was also observed in our investigations for C_12_BetC_2_Cl.

The inspection of micellization parameters of *N*-alkyl betaine ethyl ester chlorides calculated using Equations (1)–(4) collected in Table 1 shows that the maximum excess surface, *Γ*_max_, generally decreases with the increasing length of the alkyl moiety at the polar amine. For C_12_BetC_2_Cl, the maximum surface excess concentration decreases with the increase in temperature. At the same time, the minimum area, *A*_min_, increases with *n* and also with *t* for C_12_BetC_2_Cl. *Γ*_max_ of C_12_BetC_2_Cl at 25 °C is almost two times lower than those for decylbetaine, C_10_Bet, (3.1·10^−6^ mol·m^2^) [10], or dodecytrimethylammonium bromide DTAB (3.1·10^−6^ mol·m^2^) [32], when *A*_min_ is two times higher (0.53·10^−18^ m^2^ and 0.553·10^−18^ m^2^, respectively) [10,32]. The lower maximum surface excess with a higher minimum surface area for C*_n_*BetC_2_Cl than for C_10_Bet presumably arises from a steric hindrance induced by an ethyl ester group present instead of the pure carboxyl. From the ^1^H NMR investigations of micelle hydrations of amphiphilic betaine ester derivatives (CH_3_)_3_N^+^-CH_2_COOC*_n_*H_2n+1_ X^−^, when *n* = 10, 12, 14, and 16, it was found that ester group COO- behaves during micellization as if it were a CH_2_CH_2_ group [18]. According to the above, the ethyl chain may also cause the whole part of the ester to be more hydrophobic, requiring higher values of minimum area per molecule to locate at the water–air interface.

The efficiency of the surface absorption *p*C_20_, calculated for the surfactants here, is comparable with those observed for dodecytrimethylammonium bromide, DTAB, [31] or dodecytrimethylammonium chloride DTAC [38], and the effectiveness of surface tension reduction, *γ*_CMC_, is on the same level. In this study, the variation of *p*C_20_ with the increasing chain length is very weak; similarly, the temperature change has little effect on this parameter. The only difference in *p*C_20_ is between C_8_BetC_2_Cl and other C_10_BetC_2_Cl and C_12_BetC_2_Cl compounds, as shown in Table 1. The standard free energy of micellization, Δ*G_m_*, and adsorption, ∆*G_ads_*, for surfactants under study in aqueous solution (see Table 1) are negative and decrease with the alkyl chain length and with the temperature increase. They are both in values typical for cationic surfactants, such as dodecytrimethylammonium bromide, DTAB [32], or betaine ester bromides with the alkyl chain from octyl to octadecyl in the ester moiety (CH_3_)_3_N^+^-CH_2_COOC*_n_*Br^−^ [20]. The knowledge of the minimum area occupied by the amphiphilic molecule at the water–air interface, *A*_min_, can be further used for estimation of the packing parameter, *P*, according to the Tanford formula (*P* = *v*/(*l_c_*∙*A*), where *v* is the volume of the hydrophobic chain, and *l_c_* is the maximum effective length for the hydrophobic chain; *A* should be the surface area of the polar group, however, in the calculations *A*_min_ is used (due to difficulties in *A* calculations) [39]. According to equations given by Tanford [39] and *A*_min_ values taken from Table 1, the packing parameters are for all C*_n_*BetC_2_Cl below 0.3 at all temperatures, indicating, as expected, the spherical shape of the aggregates formed for the currently studied systems.

### 2.3. Apparent Molar Properties

The apparent molar quantities, *X_ϕ_*, are less clearly understood than the partial molar quantities, *X_i_*; however, *X_ϕ_* has some advantages over *X_i_*. The apparent molar quantity is related to the global quantity of solution (*V* or *K_S_*) and a molar property of pure solvent, *X*^o^, by the relation: $X=X_{w}^{o}\cdot n_{w}+X_{\phi }\cdot n_{s}$, where *X_ϕ_* is the apparent molar volume, *V_ϕ_*, or the apparent molar isentropic compressibility, *K_sϕ_*. *X_ϕ_* refers to the solute propertyhere, *N*-alkyl betaine ethyl ester chlorides; *n* is the number of moles; subscript *w* refers to water (solvent); and *s* refers to solute.

The meaning of *X_ϕ_* is similar to partial molar quantities (the contribution of the solute to the total volume or compressibility of a solution). However, it can be directly calculated from experimental density and speed of sound; the apparent molar quantity is a sum of the volume or compressibility of the solute and the changes that the solute causes in a solvent by its presence. Additionally: $X_{\phi }^{\infty }=X_{\phi }$, and $X_{i}^{\infty }=X_{\phi }^{\infty }$, where $X_{i}^{\infty }$ is the partial molar quantity at the hypothetical infinite dilution, and is equal to the apparent molar quantity at the same state, $X_{i}^{\infty }$.

Apparent molar volume can be calculated employing equation:

(5) $$V_{\phi }=\frac{M_{s}}{\rho }-\left[\frac{10^{3}\cdot \left(\rho -\rho_{w}\right)}{m\cdot \rho \cdot \rho_{w}}\right]$$

where *m* means molality, and density, *ρ*, without a subscript refers to a solution.

Apparent molar adiabatic compressibility is calculated from density and adiabatic compressibility, ($\kappa_{s}=-\frac{1}{V}\cdot {\left(\frac{\partial V}{\partial p}\right)}_{s}$), according to equations:

(6) $$\kappa_{s}=\frac{1}{\rho \cdot c^{2}}$$

(7) $$K_{S,\;\phi }=\frac{\kappa_{S}\cdot M_{s}}{\rho }-\frac{\kappa_{s,\;w}\cdot \rho -\kappa_{s}\cdot \rho_{w}}{m\cdot \rho \cdot \rho_{w}}$$

where: *ρ*—density, *c*—speed of ultrasound, *V*—volume, *p*—pressure, *S*—entropy (here adiabatic means also isentropic, when the energy transfer is reversible).

**Figure 4:**
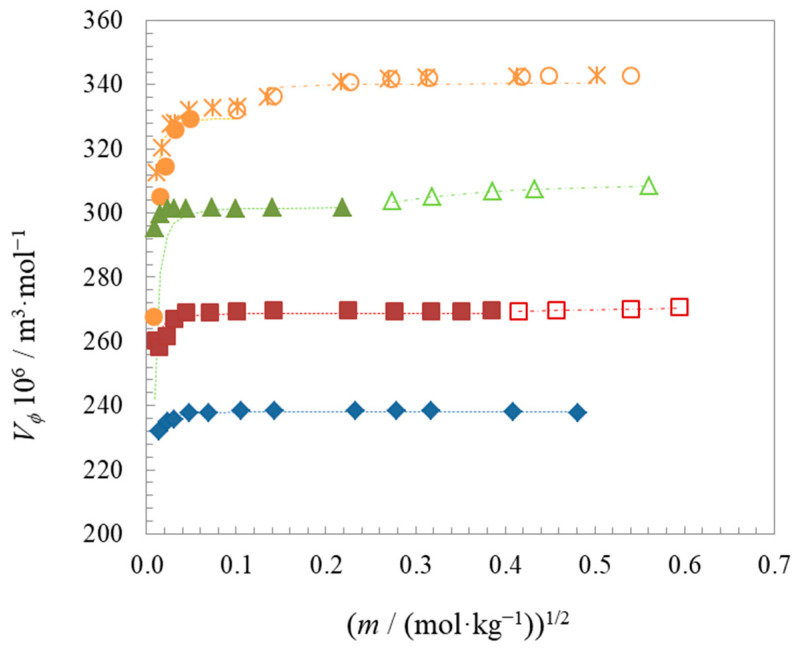
Apparent molar volume of C*_n_*BetC_2_Cl in aqueous solutions at *t* = 25 °C; points: (♦) *n* = 6, (■, □) *n* = 8, (▲, Δ) *n* = 10, (●, ○) *n* = 12 (second series), (∗) *n* = 12 (first series); filled symbols—before breakdown (at CMC), empty symbols—after breakdown (at CMC); lines—according Equation (9).

**Figure 5:**
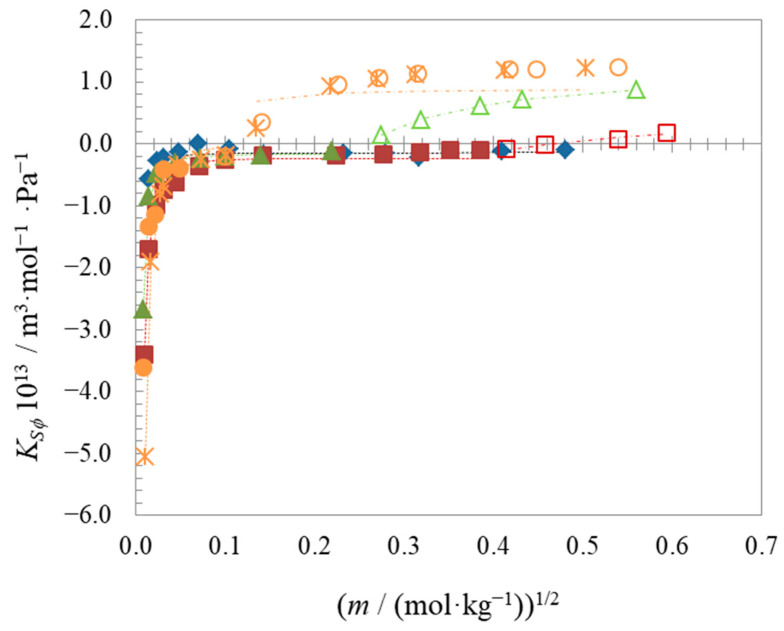
Apparent molar adiabatic compressibility of C*_n_*BetC_2_Cl in aqueous solutions at *t* = 25 °C; points: (♦) *n* = 6, (■, □) *n* = 8, (▲, Δ) *n* = 10, (●, ○) *n* = 12 (second series), (∗) *n* = 12 (first series); filled symbols—before breakdown (at CMC), empty symbols—after breakdown (at CMC); lines—according Equation (9).

Adiabatic compressibility calculated from Equation (6) based on experimental density and speed of sound in the studied solutions are tabulated in Table S4 in the Supplementary Materials. The results of calculations according to Equations (5) and (7) are listed in Figure 4 and Figure 5, Table S4, Figures S25 and S26 in the Supplementary Materials. In Table S4, the calculated expected errors of the apparent molar volumes, *δV_ϕ_*, are also reported.

The concentration dependence of apparent molar quantities $X_{\phi }$ (volume or isentropic compressibility *V* or *K_S_*), especially at a low concentration range, can be described usually by using the Redlich–Rosenfend $X_{\phi }=X_{\phi }^{\infty }+S_{X}\cdot m^{1/2}+B_{X}\cdot m$ [40] type equation at a given temperature, with *S_x_* and *B_x_* as adjustable parameters discussed often in terms of ion–ion, ion–solvent interactions. The *S_x_* parameter can also be predicted theoretically for all electrolytes of a given charge type in a given solvent and at a given temperature (according to Debye–Hückel’s theory) [41].

However, due to possible micellization processes, and for simplicity for our systems, we decided to apply another form of equations to represent *X_ϕ_*(*m*) dependences. Our starting point was a straightforward—although in this case appropriate—phase-separation model described by Rosenholm [42] according to which the volume, or compressibility change associated with micelle formation, can be represented as a difference between the apparent molar volume or compressibility of the solute regarded as a monomer in the micellar state, $X_{\phi }^{mic}$ (when *m* > CMC) and the apparent molar volume of the solute in the monomeric state, $X_{\phi }^{mon}$(when *m* < CMC):

(8) $$\Delta X_{\phi }=X_{\phi }^{mic}-X_{\phi }^{mon}$$

In order to find $X_{\phi }^{mic}$ and $X_{\phi }^{mon}$, we described separately concentration dependences of *X_ϕ_* before and after the CMC with a polynomial of the following form:

(9) $$X_{\phi }=a+b\cdot (1/m)$$

where *a* and *b* are adjustable parameters, and *a* can be regarded as $X_{\phi }^{mic}$ and $X_{\phi }^{mon}$ depending on the concentration range. Equation (9) is especially recommended for the post-micellar region [42]. Since any form of the Redlich–Rosenfend equation was not efficient enough in the presentation of the *X_ϕ_*(*m*) dependence (see Figure 4, Figure 5, Figures S24 and S25 in the Supplementary Materials), we decided to use the same form of polynomial also for the pre-micellar region, or C_6_BetC_2_Cl, in the whole regarded concentration range. Parameters of Equation (9) for the apparent molar volume and isentropic compressibility, as well as the Δ*V_ϕ_* and Δ*K_Sϕ_* change (from Equation (8)) during the micellization process for all *N*-alkyl betaine ethyl ester chlorides, are collected in Table 2.

The inspection of Figure 3 and Figure 4, Table 2, and Figures S24 and S25, and Table S4 in the Supplementary Materials indicate the increase in *V_ϕ_*, *K_Sϕ_*, $V_{\phi }^{mon}$, $V_{\phi }^{mic}$, $K_{s\phi }^{mon}$ and $K_{s\phi }^{mic}$ with the length of the alkyl moiety in the ammonium group of the *N*-alkyl betaine ethyl ester chlorides. However, the variation of $K_{s\phi }^{mon}$ and $K_{s\phi }^{mic}$ in the series C_6_–C_12_ is less noticeable. It seems that the temperature decrease causes the decrease in Δ*V_ϕ_* and Δ*K_Sϕ_*, which is intuitive and agrees with the literature reports [42,43,44,45,46]. The results found in the literature for Δ*V_ϕ_* for some surfactants (including *N*-alkyltrimethylammonium bromides, sodium or lithium alkyl sulfates with the butyl-, octyl-, decyl-, and dodecyl- moiety) are of the same order, which is very surprising, taking into account different approaches applied for calculations of $V_{\phi }^{mon}$ [42,43,44,45,46]. It should also be noticed that the proper literature values for C_10_BetC_2_Cl are $V_{\phi }^{mon}$∙10^6^ = 302.4 m^3^∙mol^−1^ and $V_{\phi }^{mic}$∙10^6^ = 309.9 m^3^∙mol^−1^ at 25 °C [22], which are very close to those obtained in this study. Finally, the positive *K_Sϕ_* for C_10_ and C_12_ after the CMC (Figure 5) are worth mentioning. Such observation supports our earlier presumption about the increasing rigidity of solutions when the density decreases, and ultrasound propagation throughout the solution is less effective.

### 2.4. Viscosity

**Figure 6:**
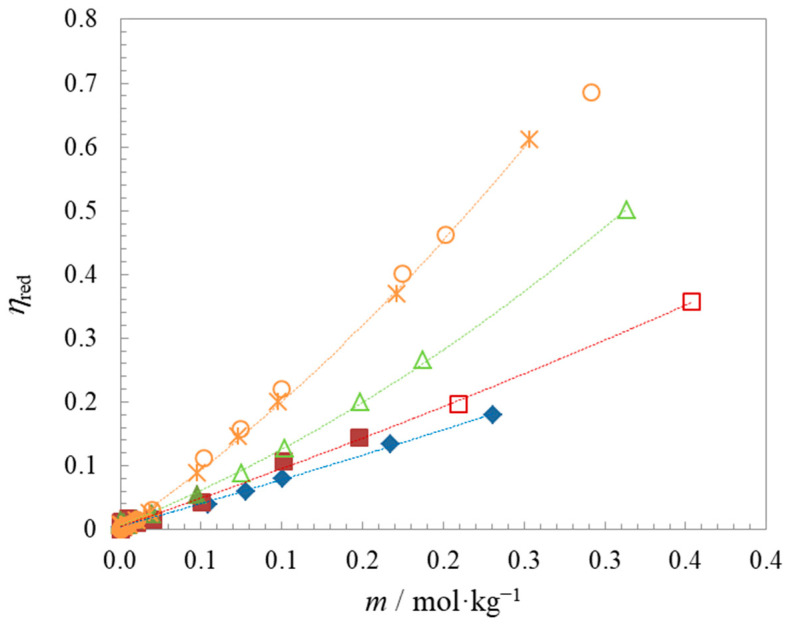
Reduced viscosity of aqueous solutions of C*_n_*BetC_2_Cl at *t* = 25 °C; experimental points: (♦) *n* = 6, (■, □) *n* = 8, (▲, Δ) *n* = 10, (●, ○) *n* = 12 (second series), (∗) *n* = 12 (first series); filled symbols—before breakdown (at CMC), empty symbols—after breakdown (at CMC) lines—according to Equation (10).

The experimental dynamic viscosity of the aqueous solution of *N*-alkyl betaine ethyl ester chlorides (C*_n_*BetC_2_Cl (*n* = 6, 8, 10, 12 (1))) at *t* = 25 °C and of C_12_BetC_2_Cl at temperatures *t* = (15–45) °C is provided in Table S2 in the Supplementary Materials. For further calculations and characterization of intermolecular interactions occurring in the investigated mixtures, the concentration dependence of viscosity of dilute electrolyte solution is the Jones–Dole equation of the form:

(10) $$\eta_{red}=A\cdot m^{1/2}+B\cdot m$$

where: $\eta_{red}=\eta_{rel}-1=\frac{\eta }{\eta_{0}}-1$, with *η* and *η*_0_ denoting mean viscosity of solution and solvent, respectively; *A* and *B* are adjustable constants of the magnitude, and the sign of the *B* coefficient can be a measure of the solute’s influence on the water structure. The sign of temperature *B* dependence is sometimes discussed in terms of the structure-breaker and structure-maker ability of ions toward a solvent. The d*B*/d*T* coefficient was reported as negative for small ions, positive for larger ones, and almost equal to zero for ions of medium radii [47,48]. *A* and *B* coefficients obtained from fitting the dynamic viscosity to Equation (10) are listed in Table 3. The reduced viscosity vs. concentration for C*_n_*BetC_2_Cl (*n* = 6, 8, 10, 12 (1)) at *t* = 25 °C is presented in Figure 6.

From Table 3, it can be seen that *A* (present in the Jones–Dole equation (Equation (10)) decreases, more or less regularly, in the series for aqueous solutions of C_6_BetC_2_Cl to C_12_BetC_2_Cl at 25 °C and changes irregularly for C_12_BetC_2_Cl with the temperature; the temperature dependence of *A* and *B* (Equation (10)) for compounds regarded here is challenging to establish, or it is very slight in magnitude; the same was observed for the surface tension and CMC calculated from different physicochemical parameters.

Positive *B* values mean an increase in the rigidity of the structure, and d*B*/d*T* ≈ 0 may indicate no significant influence of ions on the environment overall. These observations agree with the results obtained for some amphiphiles and amino acids [48]. However, a positive *B* value is attributed to larger ion sizes rather than other effects. We also presume the viscosity to be a macroscopic property, which is not sufficiently sensitive to the complex interactions occurring in surfactant solutions, especially for such low concentrations. According to Einstein’s observations, *η* is usually low (compared to solvent) for ionic surfactant solutions with globular shapes [47]. Thus, we decided to characterize this quantity as a crucial parameter in solutions/emulsion formulations used in pharmacy, for detergents and other applications.

### 2.5. Dynamic Light Scattering

**Figure 7:**
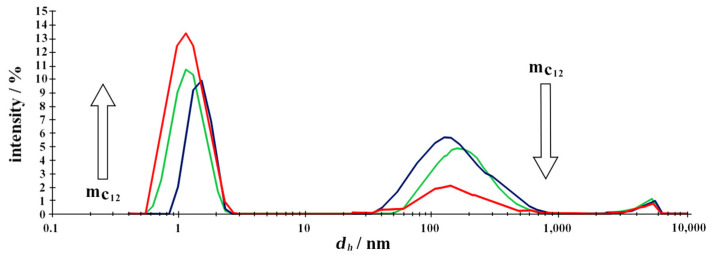
Dynamic light scattering for aqueous solutions of C_12_BetC_2_Cl at concentrations of (from the top): *m* = 3.3 × CMC (0.05 mol∙kg^−1^)—blue line, *m* = 6.7 × CMC (0.1 mol∙kg^−1^)—green line, and at *m* = 13.3 × CMC (0.2 mol∙kg^−1^)—red line; *t* = 25 °C.

Dynamic light scattering (DLS) is a valuable tool for studying the diffusion behavior of macromolecules in solution. The hydrodynamic radii or diameters can be calculated since they depend on the size and shape of macromolecules. DLS also gives information about the homogeneity of the analyzed solution in micelle size distribution. In this study, DLS measurements were performed for three solutions of C_12_BetC_2_Cl at 25 °C for concentration above the CMC, and 1M C_10_BetC_2_Cl (above the CMC) for comparison; the results are presented in Figure 7, and Table 4.

The inspection of Figure 7 and results listed in Table 4 show that in the investigated C_12_BetC_2_Cl solution just after the CMC (*m* = 3.3 × CMC (0.05 mol∙kg^−1^)), there are mainly presented two fractions of aggregates with sizes (the hydrodynamic diameters) of around 170 nm and 1.5 nm with a ratio 60:38, whereas for *m* = 13.3 × CMC (0.2 mol∙kg^−1^), smaller objects (1.2 nm) prevail over 180 nm with the ratio 78:20. Micelles larger than the two mentioned above occurred in a minimal amount (below 3%).

Our preliminary investigations also found that the smallest aggregates just appeared around the CMC, where the dynamics of changes in shape and size of micelles are very high. In contrast, in the premicellar concentrations of C_12_BetC_2_Cl, only larger objects are present in solutions: *d_h_* ≈ 30–50 nm and 200–250 nm, fluctuating with sizes and ratios. Finally, the results obtained for 1 M C_10_BetC_2_Cl allow us to conclude that the larger-size micelles are still present in more concentrated solutions (as was for 0.2 mol∙kg^−1^ C_12_BetC_2_Cl). However, micelles of smaller diameters are dominant, of around 1.2–1.4 nm, which are probably responsible for all phenomena registered for concentration dependencies of density, speed of sound, and surface tension for aqueous solutions of C_10_ and C_12_ (and sometimes C_8_) homologs.

The literature survey shows that for dodecyl betaine, C_12_Bet, with a CMC of around 2 mM, the hydrodynamic diameters of aggregates is 2.4 nm [3,7]; for dodecyl trimethylammonium bromide in the absence of other electrolytes, DTAB, the diameter of the spherical micelles is *d_h_* = 3.5 nm [49], whereas, in sodium dodecyl sulfate solution, SDS, calculated *r_h_* = 1.93–2.14 nm for concentration range 0.008–0.06 M is close to the experimental value 1.84 nm (*c* ≈ 0.07 M) [50]. Micelle radius *r*_M_, and hydrated micelle radius *r*_Mhydr_, calculated for C_10_BetC_2_Cl by Medos et al. [22] based on density measurements, isothermal titration calorimetry (ITC), and dielectric relaxation spectroscopy (DRS), were estimated as between values of 1.13–1.17 nm and 1.23–1.27 nm, respectively.

The picture of micellization obtained from DLS measurements differs from what was expected since the *d_h_* values of C_12_BetC_2_Cl micelles are 2–4 times smaller than in similar surfactant solutions. However, comparably small micelles sizes have already been reported in the literature of 1-decyl-3-methylimidazolium chlorides, whereas bromides and tetrafluoroborates, *d_h_* are larger [51]. It is also very interesting to observe larger aggregates just after the CMC in the C_12_BetC_2_Cl aqueous solution that disappear at higher values of *m*. The presence of the ethyl ester group is a steric hindrance, and apart from the repulsion between alkyldimethylammonium heads, it prevents the formation of larger aggregates in C_12_BetC_2_Cl aqueous solutions. The most puzzling is the influence of the water molecules, which may stabilize the larger aggregates above CMC to some extent, but at higher concentrations, this effect decreases. The presence of chloride anions undoubtedly completes the picture of possible interactions where they interact with positive centers located at the nitrogen atom.

The larger aggregates of amphiphiles, present in a certain amount in solutions of C_12_BetC_2_Cl at *m* = 3.3 × CMC, can also be efficient in the solubilization of some hydrophobic drugs due to having more space in their hydrophobic core [51,52,53]. For this reason, the most suitable concentrations for using investigated SAILs (C_10_ and C_12_) as co-surfactants and stabilizers, according to the micellar scheme, are above the CMC.

## 3. Materials and Method

### 3.1. Materials

1-bromohexane (98%), 1-bromooctane (99%), 1-bromodecane (98%) and 1-bromododecane (95%) were obtained from Sigma-Aldrich Co. (St. Louis, MO, USA). Dimethylamine hydrochloride (99%) was purchased from Acros Organics company (Geel, Belgium). Diethyl ether (99.5%), ethanol (99.8%), acetone (99.5%), sodium hydroxide (99%) and ethyl chloroacetate (99%) were purchased from POCH S.A. (Gliwice, Poland). Deionized water used for synthesis had a specific conductivity not higher than 0.4 μS∙cm^−1^ at 20 °C.

### 3.2. Synthesis and Characterization

Infrared spectra for powder samples were recorded using a Fourier-transform infrared (FTIR) spectrometer Jasco FT/IR-4600LE (Jasco, Tokyo, Japan). The spectra were obtained by accumulation of 59 scans with 4 cm^−1^ resolution in the region of 4000–750 cm^−1^.

Mass spectroscopy (LC-MS) for the title compounds was performed using an Agilent 1260 Infinity II LC/MSD XT system (Agilent, Waldbronn, Germany).

^1^H NMR spectra were recorded in CDCl_3_ using a Bruker Avance III 500 MHz NMR spectrometer (Bruker Switzerland AG, Fällanden, Switzerland) at 25 °C measured in CDCl_3_ (7.26 ppm). ^13^C NMR spectra were recorded in CDCl_3_ (and DMSO-D6 for the comparative spectrum of compound C_12_BetC_2_Cl) at 126 MHz. ^1^H and ^13^C NMR chemical shifts (ppm) are referenced to the either residual CDCl_3_ (δH 7.26, δC 77.1) or DMSO-D6 (δC 39.5) and J-values are given in Hz.

Melting points of the title compounds were measured with an Optimelt MPA 100 Instruments (Stanford Research Systems, Sunnyvale, CA, USA) and are uncorrected. The temperature resolution was ±0.1°C, whereas temperature reproducibility was on the level ±0.2 °C, and the heating rate was 1.0 °C/min.

Thermogravimetric analysis (TGA) of samples of a mass around 2 mg was performed in the temperature range of 15–600 °C using a Perkin Elmer Pyris 1 TGA (PerkinElmer, Waltham, MA, USA) with a heating rate of 10 °C/min in a stream of nitrogen flow (25 mL/min), whereas Differential Scanning Calorimetry (DSC) measurements were performed with aluminum sample pans under nitrogen atmosphere with a heating rate of 10 °C/min. The TGA and DSC analysis results of synthesized compounds are presented in Figures S19 and S20 in the Supplementary Materials.

The basic information on the yield of synthesis *N*-alkyl betaine ethyl ester chlorides, C*_n_*BetC_2_Cl, together with their thermal properties, is listed in Table 5.

Due to similar composition, the melting temperature range and the thermal properties of all compounds under study are very close. The range of melting temperatures of C_10_BetC_2_Cl in the literature was 93–98 °C, whereas the thermal decomposition was characterized by a single temperature point only at 130 °C [22]. The TGA and DSC curves recorded in this study (Figures S19 and S20, in the Supplementary Materials) are similar to those reported by Medos et al. [22].

#### 3.2.1. *General Procedure for the Synthesis of Dimethyl Alkyl Amines (Step 1—See Scheme 2)*

Dimethyl alkyl amines with unbranched alkyl chain C_6_, C_8_, C_10_, and C_12_ were synthesized by reacting dimethylamine (prepared in situ from dimethylamine hydrochloride and sodium hydroxide) with the appropriate 1-bromoalkane (C*_n_*H_2*n*+1_Br with *n* = 6, 8, 10, and 12) according to the protocols described by Menger and Peresypkin [54] and Zhang et al. [55] with some modifications. Briefly, dimethylamine hydrochloride (90 mmol) and 100 mL of ethanol–water mixed solvent (*v*/*v* = 95:5) were added in a 250 mL flask and stirred to dissolve. Then, a solution of NaOH (100 mmol in 10 mL water) and appropriate alkyl bromide (30 mmol) were added, and the mixture was stirred at 100 °C under reflux for 24 h. After the solvent was removed by vacuum evaporation, 80 mL of a 10% sodium hydroxide solution was added and stirred. The product was then extracted three times with 30 mL of diethyl ether, dried (MgSO_4_), concentrated in vacuo, and distilled under reduced pressure to afford liquid products. ^1^H NMR spectra are given in the Supplementary Materials (Figures S1–S4). Characteristics of materials used for the synthesis of alkyl betaine esters in this study are collected in Table S1 in the Supplementary Materials.

**Scheme 2:**
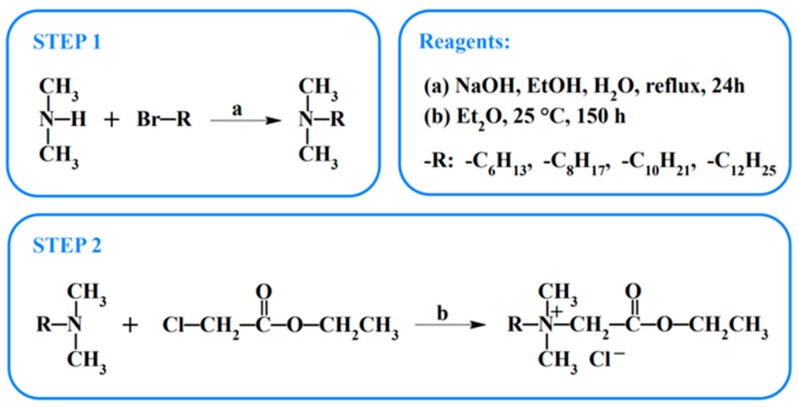
Synthesis of *N*-alkyl betaine ethyl ester chlorides, C*_n_*BetC_2_Cl (*n* = 6, 8, 10, 12).

*N,N-dimethylhexan-1-amine.*

Obtained in yield of 61% as a colorless liquid. ^1^H NMR (500 MHz, CDCl_3_) δ: 2.30–2.25 (m, 2H, CH_2_N), 2.24 (s, 6H, N(CH_3_)_2_) 1.51–1.42 (m, 2H, NCH_2_CH_2_), 1.33–1.24 (m, 6H, (CH_2_)_3_(hexyl)), 0.92–0.84 (m, 3H, CH_3_(hexyl)). The ^1^H NMR report is corresponding with the reference [56]; the product was used in the next step of the synthesis.

*N,N-dimethyloctan-1-amine.*

Obtained in yield of 91% as a colorless liquid. ^1^H NMR (500 MHz, CDCl_3_) δ: 2.32–2.21 (m, 8H, CH_2_N(CH_3_)_2_), 1.51–1.43 (m, 2H, NCH_2_CH_2_), 1.33–1.21 (m, 10H, (CH_2_)_5_(octyl)), 0.90–0.84 (m, 3H, CH_3_(octyl)). The ^1^H NMR report is corresponding with the reference [57]; the product was used in the next step of the synthesis.

*N,N-dimethyldecan-1-amine.*

Obtained in yield of 90% as a colorless liquid. ^1^H NMR (500 MHz, CDCl_3_) δ: 2.30–2.25 (m, 2H, CH_2_N), 2.24 (s, 6H, N(CH_3_)_2_) 1.50–1.43 (m, 2H, NCH_2_CH_2_), 1.33–1.23 (m, 14H, (CH_2_)_7_(decyl)), 0.87 (t, *J* = 6.9 Hz, 3H, CH_3_(decyl)). The ^1^H NMR report is corresponding with the reference [57]; the product was used in the next step of the synthesis.

*N,N-dimethyldodecan-1-amine.*

Obtained in yield of 93% as a colorless liquid. ^1^H NMR (500 MHz, CDCl_3_) δ: 2.41–2.36 (m, 2H, CH_2_N), 2.33 (s, 6H, N(CH_3_)_2_) 1.59–1.46 (m, 2H, NCH_2_CH_2_), 1.33–1.20 (m, 18H, (CH_2_)_9_(dodecyl)), 0.87 (t, *J* = 6.9 Hz, 3H, CH_3_(dodecyl)). The ^1^H NMR report is corresponding with the reference [57]; the product was used in the next step of the synthesis.

#### 3.2.2. *General Procedure for the Synthesis of N-alkyl Betaine Ethyl Ester Chlorides (Step 2—See Scheme 2)*

*N*-alkyl betaine ethyl ester chlorides were obtained by the reacting of ethyl chloroacetate with appropriate *N*-alkyldimethylamine (C*_n_*H_2*n*+1_N(CH_3_)_2_ with *n* = 6, 8, 10, 12), according to the protocol described by Abd El-Lateef et al. [58] with some modifications. Briefly, *N*,*N*-dimethylalkylamine (10 mmol), ethyl chloroacetate (10 mmol), and 30 mL of diethyl ether were placed in a single-neck flask equipped with a magnetic stir bar. The reaction mixture was stirred continuously at 25 °C for 150 h. The resulting precipitate was filtered off, washed with diethyl ether and dried under reduced pressure. In the case of products of insufficient purity (NMR analysis), we used crystallization from the acetone-diethyl ether system. The ^1^H NMR, ^13^C NMR, FT-IR (ATR), and LR-ESI-MS spectra of products are given in the Supplementary Materials (Figures S5–S12).

*N-(2-ethoxy-2-oxoethyl)-N,N-dimethylhexan-1-aminium chloride* (C_6_BetC_2_Cl).

Obtained in yield of 85% as a hygroscopic white solid (m.p.: 95.6–96.4 °C). ^1^H NMR (500 MHz, CDCl_3_) δ: 0.83–0.91 (m (t-like), 3H, CH_3_(hexyl)), 1.18–1.42 (m, 9H, (CH_2_)_3_(hexyl) and CH_3_(ethyl)), 1.64–1.77 (m, 2H, CH_2_CH_2_N), 3.61 (s, 6H, 2NCH_3_), 3.70–3.81 (m, 2H, CH_2_CH_2_N), 4.23 (q, *J* = 7.2 Hz, 2H, CH_2_O), 5.02 (s, 2H, NCH_2_COO). ^13^C NMR (126 MHz, CDCl_3_) δ: 13.56, 13.64, 22.03, 22.48, 25.48, 30.85, 51.16, 60.90, 62.23, 64.05, 164.71. FTIR (ATR) *ν*/cm^−1^: 2925, 2858, 1742, 1468, 1415, 1229, 1032, 901. LR-ESI-MS (*m*/*z*, 251.79 g/mol), calculated for [M-Cl^−^]: 216.195, found: 216.1.

*N-(2-ethoxy-2-oxoethyl)-N,N-dimethyloctan-1-aminium chloride* (C_8_BetC_2_Cl).

Obtained in yield of 91% as a hygroscopic white solid (m.p.: 83.0–84.6 °C). ^1^H NMR (500 MHz, CDCl_3_) δ: 0.80–0.89 (m (t-like), 3H, CH_3_(octyl)), 1.16–1.38 (m, 13H, (CH_2_)_5_(octyl) and CH_3_(ethyl)), 1.64–1.74 (m, 2H, CH_2_CH_2_N), 3.60 (s, 6H, 2NCH_3_), 3.70–3.80 (m, 2H, CH_2_CH_2_N), 4.22 (q, *J* = 7.1 Hz, 2H, CH_2_O), 5.01 (s, 2H, NCH_2_COO). ^13^C NMR (126 MHz, CDCl_3_) δ: 13.64, 13.72, 22.22, 22.54, 25.84, 28.66, 28.72, 31.27, 51.17, 60.90, 62.21, 64.02, 164.72. FTIR (ATR) *ν*/cm^−1^: 2923, 2856, 1740, 1469, 1412, 1215, 1027, 901. LR-ESI-MS (*m*/*z*, 279.86 g/mol), calculated for [M-Cl^−^]: 244.227, found: 244.2.

*N-(2-ethoxy-2-oxoethyl)-N,N-dimethyldecan-1-aminium chloride* (C_10_BetC_2_Cl).

Obtained in yield of 93% as a hygroscopic white solid (m.p.: 93.8–95.0 °C). ^1^H NMR (500 MHz, CDCl_3_) δ: 0.85 (t, *J* = 7.0 Hz, 3H, CH_3_(decyl)), 1.12–1.40 (m, 17H, (CH_2_)_7_(decyl) and CH_3_(ethyl)), 1.64–1.74 (m, 2H, CH_2_CH_2_N), 3.60 (s, 6H, 2NCH_3_), 3.70–3.79 (m, 2H, CH_2_CH_2_N), 4.22 (q, *J* = 7.2 Hz, 2H, CH_2_O), 5.01 (s, 2H, NCH_2_COO). The ^1^H NMR spectra is corresponding with the reference [22]. ^13^C NMR (126 MHz, CDCl_3_) δ: 13.70, 13.83, 22.36, 22.61, 25.90, 28.84, 28.94, 29.07, 29.11, 31.54, 51.23, 60.96, 62.27, 64.05, 164.80. FTIR (ATR) *ν*/cm^−1^: 2921, 2852, 1740, 1468, 1231, 1211, 1028, 903. LR-ESI-MS (*m*/*z*, 307.90 g/mol), calculated for [M-Cl^−^]: 272.258, found: 272.2.

*N-(2-ethoxy-2-oxoethyl)-N,N-dimethyldodecan-1-aminium chloride* (C_12_BetC_2_Cl).

Obtained in yield of 93% as a hygroscopic white solid (m.p.: 87.9–89.8 °C). ^1^H NMR (500 MHz, CDCl_3_) δ: 0.85 (t, *J* = 6.9 Hz, 3H, CH_3_(dodecyl)), 1.14–1.40 (m, 21H, (CH_2_)_9_(dodecyl) and CH_3_(ethyl)), 1.64–1.76 (m, 2H, CH_2_CH_2_N), 3.60 (s, 6H, 2NCH_3_), 3.71–3.81 (m, 2H, CH_2_CH_2_N), 4.22 (q, *J* = 7.1 Hz, 2H, CH_2_O), 5.01 (s, 2H, NCH_2_COO). ^13^C NMR (126 MHz, CDCl_3_) δ: 13.72, 13.88, 22.42, 22.64, 25.93, 28.87, 29.06, 29.10, 29.18, 29.32 (2C), 31.64, 51.26, 60.99, 62.29, 64.06, 164.83. ^13^C NMR (126 MHz, DMSO-D6) δ 13.73, 13.84, 21.74, 22.05, 25.68, 28.41, 28.69, 28.76, 28.91, 28.98, 29.01, 31.27, 50.59, 60.30, 61.72, 63.94, 165.04. FTIR (ATR) *ν*/cm^−1^: 2922, 2851, 1742, 1469, 1213, 1030, 903. LR-ESI-MS (*m*/*z*, 335.95 g/mol), calculated for [M-Cl^−^]: 300.289, found: 300.3.

### 3.3. Sample Preparation

Directly before solution preparation, all solids of C*_n_*BetC_2_Cl were dried for a minimum of 24 h under vacuum at 30 °C. Aqueous solutions of amphiphiles were prepared by weight from the stock solution using deionized water with a specific conductivity not higher than 0.4 μS∙cm^−1^ (at 20 °C). For this purpose, an analytical balance OHAUS Discovery was used with a repeatability of weighing at the level ±0.1 mg. All solutions were filtered and degassed in an ultrasonic cleaner prior to measurements. The pH of solutions of C*_n_*BetC_2_Cl was between 3 and 5 at 25 °C. Thus, we assumed that hydrolysis of esters did not occur under this condition. In addition, for the exemplary one-year-old sample of C_10_BetC_2_Cl, the ^1^H NMR spectrum was again reordered and compared with those prepared for the sample directly after synthesis, and no hydrolysis product was observed.

### 3.4. Density and Speed of Sound

Density, *ρ*, and speed of sound, *c*, in aqueous solutions of *N*-alkyl betaine ethyl ester chlorides were measured simultaneously using a digital vibrating tube densitometer and the speed-of-sound analyzer Anton Paar DSA 5000 M (Anton Paar, Graz, Austria), where the repeatability declared by the supplier for the density is 1∙10^−6^ kg∙m^−3^ and for the speed of sound, 0.1 m∙s^−1^ (according to ISO 5725-2 [59]). The uncertainties are: for the density measurements ± 2∙10^−5^ kg∙m^−3^, for the speed of sound ± 0.1 m∙s^−1^, and for temperature measurements ± 0.01 °C.

### 3.5. Surface Tension

The surface tension, *γ*, for solutions under test was measured with a Krüss DSA 100 (Krüss GmbH, Hamburg, Germany) tensiometer using the Pendant Drop technique (with Advance Software 1.13.0). The reproducibility of the surface tension was at the level ± 0.2 mN∙m^−1^. The uncertainty for temperature measured by the Pt100 thermometer placed inside the measuring cell was ±0.1 °C. All further details for this apparatus and the measurements have been previously reported [60,61,62].

### 3.6. Viscosity

The dynamic viscosity, *η*, was obtained directly from a rolling ball micro viscometer Lovis 2000 ME (Anton Paar, GmbH, Graz, Austria) with a 1.59 mm capillary. Deionized water with a specific conductivity not higher than 0.4 μS∙cm^−1^ (at *t* = 20 °C) was used for calibration. The uncertainty of temperature measurements was ±0.02 °C. The viscosity repeatability (based on the standard deviation) and accuracy reported by the manufacturer are 0.1 and 0.5%, respectively.

### 3.7. Dynamic Light Scattering

The particle diameter was measured using Malvern’s Zetasizer Nano ZS particle size analyzer (Malvern Panalytical Ltd., Malvern, UK). The device uses a He–Ne laser with a wavelength of 633 nm as a light source. Using dynamic light scattering (DLS) the analyzer determines the test particles’ diameter by measuring the sample particles’ Brownian motion velocity, which is then converted to particle diameter using the Stokes–Einstein equation: $d_{h}=\frac{kT}{3\pi \eta D}$, where *d_h_*—hydrodynamic diameter, *k*—Boltzmann’s constant, *T*—absolute temperature, *η*—viscosity of the diluent, *D*—diffusion coefficient. Zetasizer also uses the Non-Invasive Back-Scatter technique (NIBS), which involves recording back-scattered light at an angle of 173°. Measurements were carried out at 25 °C using polystyrene cuvettes with an optical path of 1 cm. The size range of the measured particles was from 0.3 nm to 10 μm, with an accuracy ± 2%.

## 4. Conclusions

The investigations of properties of *N*-alkyl betaine ethyl ester chlorides, C*_n_*BetC_2_Cl (from C_6_ to C_12_) in aqueous solutions show significant differences, manifesting in the absence or presence of the breakdown in the concentration dependences of density, speed of sound, and surface tension. The increase in the alkyl chain length as the hydrophobic part of alkyl betaine ethyl ester chloride supports the molecules’ ability to participate in the micellization process. All investigated compounds show significant surface activity, which allows them to be used for effective and efficient reduction in surface tension. The critical micelle concentration, CMC, of all presented SAILs determined using the concentration dependence of a few physicochemical parameters (from the intersection of polynomials used for *m* before and after CMC) is observed at higher concentrations than other alkyl betaine derivatives. It is comparable with the CMC of typical tetraalkylammonium salts and can be carefully controlled by the elongation of the alkyl chain in the amine. For C_8_, the CMC can be obtained only from *γ*(log*m*) dependences; density and speed of sound were less sensitive for micellization processes. For C_6_, one may expect micellization, presumably at higher concentrations, which were out of the range investigated here and beyond the interest for use in reliable applications.

From the micellization parameters, it can be concluded that aggregation of C*_n_*BetC_2_Cl is a favorable process (Δ*G_m_*, Δ*G_ads_* < 0, the efficiency of the surface absorption p*C*_20_ and *γ*_CMC_ see Table 1), as for many amphiphiles. Some other parameters, such as low values of maximum excess surface, $\Gamma_{max}$, and large minimum area, *A*_min_, compared with derivatives of amines or alkyl betaines, indicate the specific orientation of C*_n_*BetC_2_Cl molecules at the liquid–air interface caused by the steric hindrance in the hydrophobic parts. This fact can be used in some processes where the design and obtaining of thin layers on the surface are required.

The result of dynamic light scattering was especially interesting, as it showed the most probable sizes of aggregates present for different C_12_BetC_2_Cl concentrations. The most important observation is the lack of the smallest micelles below the CMC and their predominant presence at higher concentrations of amphiphiles. It allows us to conclude that the best concentration range for using the investigated SAILs (C_10_ and C_12_) as co-surfactants and stabilizers according to the micellar scheme is above the CMC (*m* ≈ 3.3 × CMC) of these compounds.

## Figures and Tables

**Table 1:** Critical micelle concentration (CMC) values from surface tension, density, and speed of ultrasound and adsorption parameters of C*_n_*BetC_2_Cl (*n* = 8, 10, 12 (1) (2)) in aqueous solutions at *t* = 25 °C and of C_12_BetC_2_Cl in aqueous solutions at *t* = (15–45) °C, with a step of 10 °C.

Compound	*t*	Surface Tension	Density	Speed of Sound	$\gamma_{CMC}$	*Γ*_max_∙10^6^	*A*_min∙_10^18^	*p*C_20_	$∆G_{m}$	$∆G_{ads}$
	°C	CMC/mol∙kg^−1^			mN∙m^−1^	mol∙m^−2^	m^2^		kJ∙mol^−1^	kJ∙mol^−1^
C_8_BetC_2_Cl	25	0.154	-	-	37.8	1.91	0.87	1.5	−14.6	−32.4
C_10_BetC_2_Cl	25	0.053	0.058	0.059	37.2	1.74	0.95	2.0	−17.2	−37.1
C_12_BetC_2_Cl (1)	25	0.012	0.010	0.013	37.2	1.85	0.90	2.6	−20.9	−39.6
C_12_BetC_2_Cl	15	0.014	0.013	0.013	38.1	1.60	1.04	2.6	−20.2	−42.2
C_12_BetC_2_Cl (2)	25	0.012	0.012	0.012	38.1	1.72	0.99	2.6	−20.7	−40.9
C_12_BetC_2_Cl	35	0.014	0.012	0.012	36.6	1.53	1.09	2.7	−21.4	−43.6
C_12_BetC_2_Cl	45	0.013	0.012	0.012	36.1	1.44	1.15	2.6	−22.1	−44.9

**Table 2:** Volumetric and isentropic compressibility properties of the micellization of *N*-alkyl betaine ethyl ester chlorides, C*_n_*BetC_2_Cl (for *n* = 6, 8, 10 and 12 (1)) in aqueous solutions calculated by fitting Equation (9) to the concentration dependence of the apparent quantities and by using Equation (8) at *t* = 25 °C, and for C_12_BetC_2_Cl at temperatures *t* = (15–45) °C, with a step of 10 °C.

Compound	*t*	$V_{\phi }^{mon}$∙10^6^	$V_{\phi }^{mic}$∙10^6^	$\Delta V_{\phi }$∙10^6^	$K_{S\phi }^{mon}$∙10^13^	$K_{S\phi }^{mic}$∙10^13^	$\Delta K_{S\phi }$∙10^13^
	°C	(m^3^∙mol^−1^)	(m^3^∙mol^−1^)	(m^3^∙mol^−1^)	(m^3^·mol^−1^∙Pa^−1^)	(m^3^·mol^−1^∙Pa^−1^)	(m^3^·mol^−1^∙Pa^−1^)
C_6_BetC_2_Cl	25	238.11 ± 0.17	- ^a^	- ^a^	−0.140 ± 0.019	- ^a^	- ^a^
C_8_BetC_2_Cl	25	268.77 ± 0.74	271.36 ± 0.54	2.6	−0.229 ± 0.039	0.400 ± 0.059	0.63
C_10_BetC_2_Cl	25	301.767 ± 0.071	309.953 ± 0.033	8.2	−0.161 ± 0.028	1.1073 ± 0.0058	1.3
C_12_BetC_2_Cl (1)	25	329.7 ± 1.3	340.5 ± 1.3	11	−0.008 ± 0.083	0.89 ± 0.19	0.9
C_12_BetC_2_Cl	15	325.3 ± 2.0	340.331 ± 0.054	15	−0.82 ± 0.17	1.2004 ± 0.0095	2.0
C_12_BetC_2_Cl (2)	25	330.0 ± 1.6	343.425 ± 0.050	13	−0.272 ± 0.076	1.3143 ± 0.0066	1.6
C_12_BetC_2_Cl	35	337.14 ± 0.38	346.416 ± 0.049	9.3	0.090 ± 0.045	1.4271 ± 0.0045	1.3
C_12_BetC_2_Cl	45	343.2 ± 4.3	349.369 ± 0.050	6.2	0.450 ± 0.076	1.5400 ± 0.0035	1.1

^a^ Not observed in the investigated concentration range.

**Table 3:** Coefficients of Equation (10) obtained from viscosity–concentration dependence for aqueous solutions of *N*-alkyl betaine ethyl ester chlorides, C*_n_*BetC_2_Cl (for *n* = 6, 8, 10, 12 (1)) at *t* = 25 °C, and for C_12_BetC_2_Cl at temperatures *t* = (15–45) °C, with a step of 10 °C.

Compound	*t*/°C	*A*/kg^1/2^∙mol^−1/2^	*B*/kg∙mol^−1^
C_6_BetC_2_Cl	25	0.03 ± 0.02	0.72 ± 0.05
C_8_BetC_2_Cl	25	−0.01 ± 0.03	1.01 ± 0.06
C_10_BetC_2_Cl	25	−0.16 ± 0.04	1.84 ± 0.10
C_12_BetC_2_Cl (1)	25	−0.20 ± 0.04	2.76 ± 0.10
C_12_BetC_2_Cl	15	−0.18 ± 0.03	2.77 ± 0.07
C_12_BetC_2_Cl (2)	25	−0.10 ± 0.01	2.54 ± 0.03
C_12_BetC_2_Cl	35	−0.12 ± 0.01	2.55 ± 0.03
C_12_BetC_2_Cl	45	−0.14 ± 0.01	2.53 ± 0.03

**Table 4:** Analysis of the particle size distribution.

Compound	*m*/(mol·kg^−1^)	*dh*_1_/nm	*dh*_2_/nm	*dh*_3_/nm	Peak 1 Area/%	Peak 2 Area/%	Peak 3 Area/%
C_10_BetC_2_Cl	1.0 ^a^	1.4	863	2871	75.6	23.2	1.2
C_12_BetC_2_Cl	0.05	1.5	173	3849	37.6	59.8	2.5
C_12_BetC_2_Cl	0.1	1.2	194	4647	53.6	43.5	2.8
C_12_BetC_2_Cl	0.2	1.2	178	3592	77.7	19.7	2.2

^a^ 1 mol∙dm^−3.^

**Table 5:** Characteristics of C*_n_*BetC_2_Cl.

Compound	*M*/g∙mol^−1^	Yield/%	*t*_melting_/°C	*t*_decomposition_/°C	
				*t*_5%_onset	*t*_50%_onset
C_6_BetC_2_Cl	251.79	85	95.6–96.4	142.25	183.58
C_8_BetC_2_Cl	279.85	91	83.0–84.6	144.71	179.87
C_10_BetC_2_Cl	307.90	93	93.8–95.0	146.33	180.99
C_12_BetC_2_Cl	335.95	93	87.9–89.8	139.41	181.47

## Data Availability

Data are contained within the article and Supplementary Materials.

## Supplementary Materials

The following Supplementary Materials can be downloaded at: https://www.mdpi.com/article/10.3390/molecules29081844/s1, Figures S1–S4: ^1^H NMR spectra of dimethyl alkyl amines; Figures S5–S10: ^1^H NMR and ^13^C NMR spectra of *N*-alkylbetaine ethyl ester chlorides; Figures S11–S14: FTIR spectra of *N*-alkylbetaine ethyl ester chlorides; Figures S15–S18: LR-MS spectra of *N*-alkylbetaine ethyl ester chlorides; Figure S19: DSC of the *N*-alkylbetaine ethyl ester chlorides, C*_n_*BetC_2_Cl; Figure S20: TGA diagrams for the *N*-alkylbetaine ethyl ester chlorides, C*_n_*BetC_2_Cl; Table S1: Characteristics of materials used for the synthesis of alkylbetaine esters in this study; Table S2: Density, speed of sound, surface tension and dynamic viscosity of aqueous solutions of *N*-alkylbetaine ethyl ester chlorides, C*_n_*BetC_2_Cl (for *n* = 6, 8, 10 and 12) at 298.15 K, and for C_12_BetC_2_Cl at temperatures *t* = (15–45) °C, with a step of 10 °C; Table S3: Coefficients of equations: $y=\sum_{i=0}^{n=2}y_{i}\cdot m^{i}$ describing concentration dependence of density, *ρ*, and speed of sound, *c*, and: γ=a+b·log⁡m for surface tension, *γ*, of aqueous solutions of *N*-alkyl betaine ethyl esters chlorides, C*_n_*BetC_2_Cl (for *n* = 6, 8, 10, 12 (1)) at 25 °C, and for C_12_BetC_2_Cl at temperatures *t* = (15–45) °C, with a step of 10 K, together with the mean deviations from the regression line: *δρ*, *δc*, *δγ* and CMC (if attainable) calculated based on the intersection of the curves before and after CMC; for density and speed of sound there is one equation for C_6_BetC_2_Cl and C_8_BetC_2_Cl, and two independent equations were found, before and after CMC for C_10_BetC_2_Cl and C_12_BetC_2_Cl; Table S4: Apparent molar volume, *V_ϕ_*, adiabatic compressibility, *κ_s_*, and apparent molar compressibility, *K_Sϕ_* of *N-*alkyl betaine ethyl ester chlorides, C*_n_*BetC_2_Cl (for *n* = 6, 8, 10, 12 (1)) in aqueous solutions at 25 °C, and for C_12_BetC_2_Cl at temperatures *t* = (15–45) °C with a step of 10 °C; Figure S21: Density of aqueous solutions of C_12_BetC_2_Cl; Figure S22: Speed of sound in aqueous solutions of C_12_BetC_2_Cl; Figure S23: Comparison of density of aqueous solutions of C_10_BetC_2_Cl at 25 °C; Figure S24: Surface tension of aqueous solutions of C_12_BetC_2_Cl; Figure S25: Apparent molar volume of C_12_BetC_2_Cl aqueous solutions; Figure S26: Apparent molar compressibility of C_12_BetC_2_Cl aqueous solutions; Figure S27: Reduced viscosity of aqueous solutions of C_12_BetC_2_Cl.
