# Supplementary material for: A Study of the Micellar Formation of N-Alkyl Betaine Ethyl Ester Chlorides Based on the Physicochemical Properties of Their Aqueous Solutions
Source: Molecules. 2024 Apr 18;29(8):1844. doi: 10.3390/molecules29081844 (PMC11053790; doi:10.3390/molecules29081844)
Supplement: Supplementary file 1 [file molecules-29-01844-s001.zip › molecules-2941382-supplementary.pdf]

Supplementary Materials

A study of the micellar formation of *N*-alkyl betaine ethyl ester chlorides based on physicochemical properties of their aqueous solutions

Monika Geppert-Rybczyńska [1*], Anna Mrozek- Wilczkiewicz [2,3], Patrycja Rawicka [2] and Piotr Bartczak [4]

[1] *Institute of Chemistry, University of Silesia, Szkolna 9, 40-006 Katowice, Poland*

[2] *August Chełkowski Institute of Physics, University of Silesia, 75 Pułku Piechoty 1, 41-500 Chorzów, Poland*

[3] *Department of Systems Biology and Engineering, Silesian University of Technology, Akademicka 16, 44-100 Gliwice, Poland*

[4] *Centre for Materials and Drug Discovery, Institute of Chemistry, University of Silesia, Szkolna 9, 40-006 Katowice, Poland*

Table of contents

Figures S1-S4: ^{1}H NMR spectra of **dimethyl alkyl amines** pages S3-S4

Figures S5-S10: ^{1}H NMR and ^{13}C NMR spectra of ***N*-alkylbetaine ethyl ester chlorides** pages S5-S9

Figures S11-S14: FTIR spectra of ***N*-alkylbetaine ethyl ester chlorides** pages S10-S11

Figures S15- S18: LR-MS spectra of ***N*-alkylbetaine ethyl ester chlorides** pages S12-S13

Figure 19: DSC of the ***N*-alkylbetaine ethyl ester chlorides**, C_nBetC$_2$Cl page S14

Figure 20: TGA diagrams for the ***N*-alkylbetaine ethyl ester chlorides**, C_nBetC$_2$Cl page S14

Table S1: Characteristics of materials used for the synthesis of alkylbetaine esters in this study page S15

Table S2: Density, speed of sound, surface tension and dynamic viscosity of aqueous solutions of *N*-alkylbetaine ethyl esters chlorides, C_nBetC$_2$Cl (for n = 6, 8, 10 and 12) at 298.15 K, and for C_{12}BetC$_2$Cl at temperatures t = (15 – 45) °C, with a step of 10 °C

pages S15-S18

Table S3: Coefficients of equations: $y = \sum_{i=0}^{n=2} y_i \cdot m^i$ describing concentration dependence of density, ρ, and speed of sound, c, and: $\gamma = a + b \cdot \log\ m$ for surface tension, γ, of aqueous solutions of *N*-alkyl betaine ethyl esters chlorides, C_nBetC$_2$Cl (for n = 6, 8, 10, 12 (1)) at 25 °C, and for C$_{12}$BetC$_2$Cl at temperatures t = (15 - 45) °C, with a step of 10 K, together with the mean deviations from the regression line: $\delta\rho$, δc, $\delta\gamma$ and CMC (if attainable) calculated based on the intersection of the curves before and after CMC; for density and speed of sound there is one equation for C$_6$BetC$_2$Cl and C$_8$BetC$_2$Cl, and two independent equations were found, before and after CMC for C$_{10}$BetC$_2$Cl and C$_{12}$BetC$_2$Cl.

pages S18-S21

Table S4: Apparent molar volume, V_ϕ, adiabatic compressibility, κ_S, and apparent molar compressibility, $K_{S\phi}$ of *N*-alkyl betaine ethyl esters chlorides, C_nBetC$_2$Cl (for n = 6, 8, 10, 12 (1)) in aqueous solutions at 25 °C, and for C$_{12}$BetC$_2$Cl at temperatures t = (15 – 45) °C with a step of 10 °C

pages S22-S24

Figure S21: Density of aqueous solutions of C$_{12}$BetC$_2$Cl page S25

Figure S22: Speed of sound in aqueous solutions of C$_{12}$BetC$_2$Cl page S25

Figure S23: Comparison of density of aqueous solutions of C$_{10}$BetC$_2$Cl at 25 °C page S26

Figure S24: Surface tension of aqueous solutions of C$_{12}$BetC$_2$Cl page S26

Figure S25: Apparent molar volume of C$_{12}$BetC$_2$Cl aqueous solutions page S27

Figure S26: Apparent molar compressibility of C$_{12}$BetC$_2$Cl aqueous solutions page S27

Figure S27: Reduced viscosity of aqueous solutions of C$_{12}$BetC$_2$Cl page S28

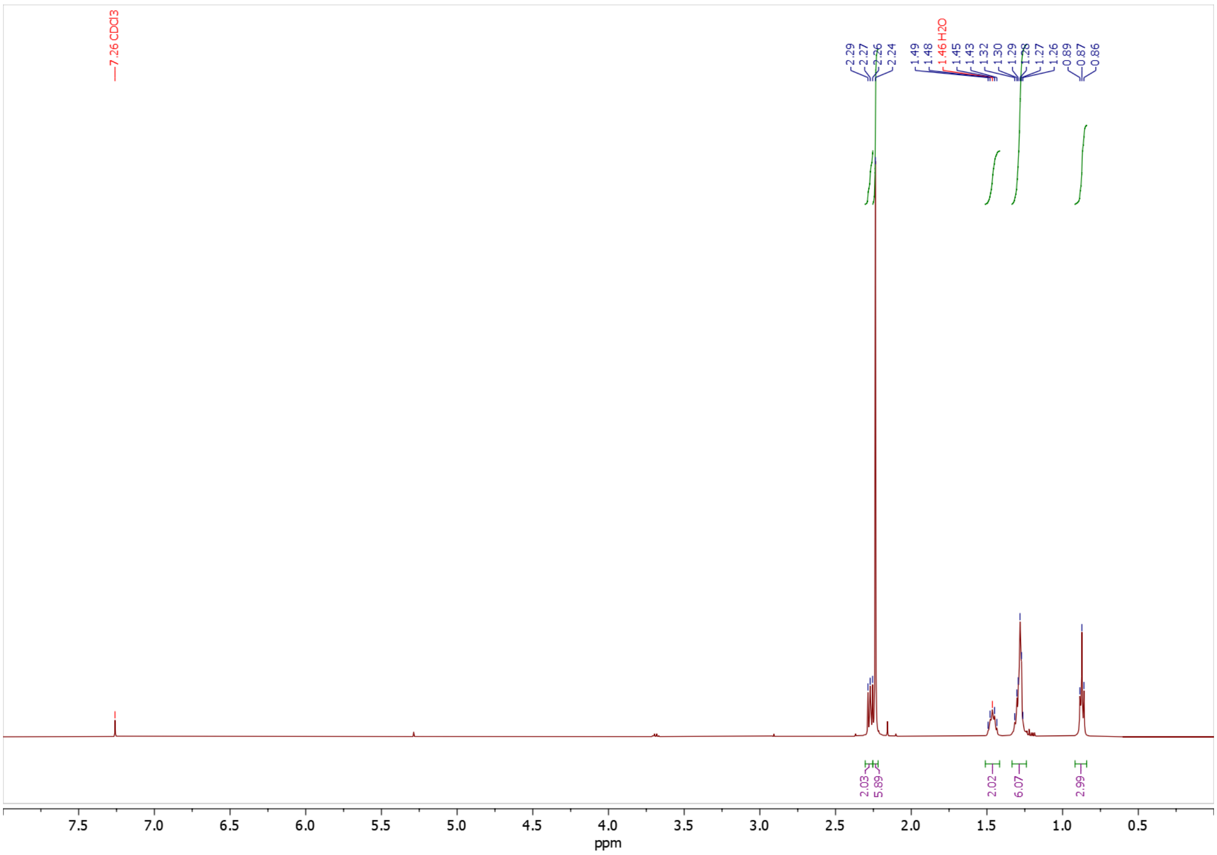

Figure S1. ^{1}H NMR spectra of *N*,*N*-dimethylhexan-1-amine

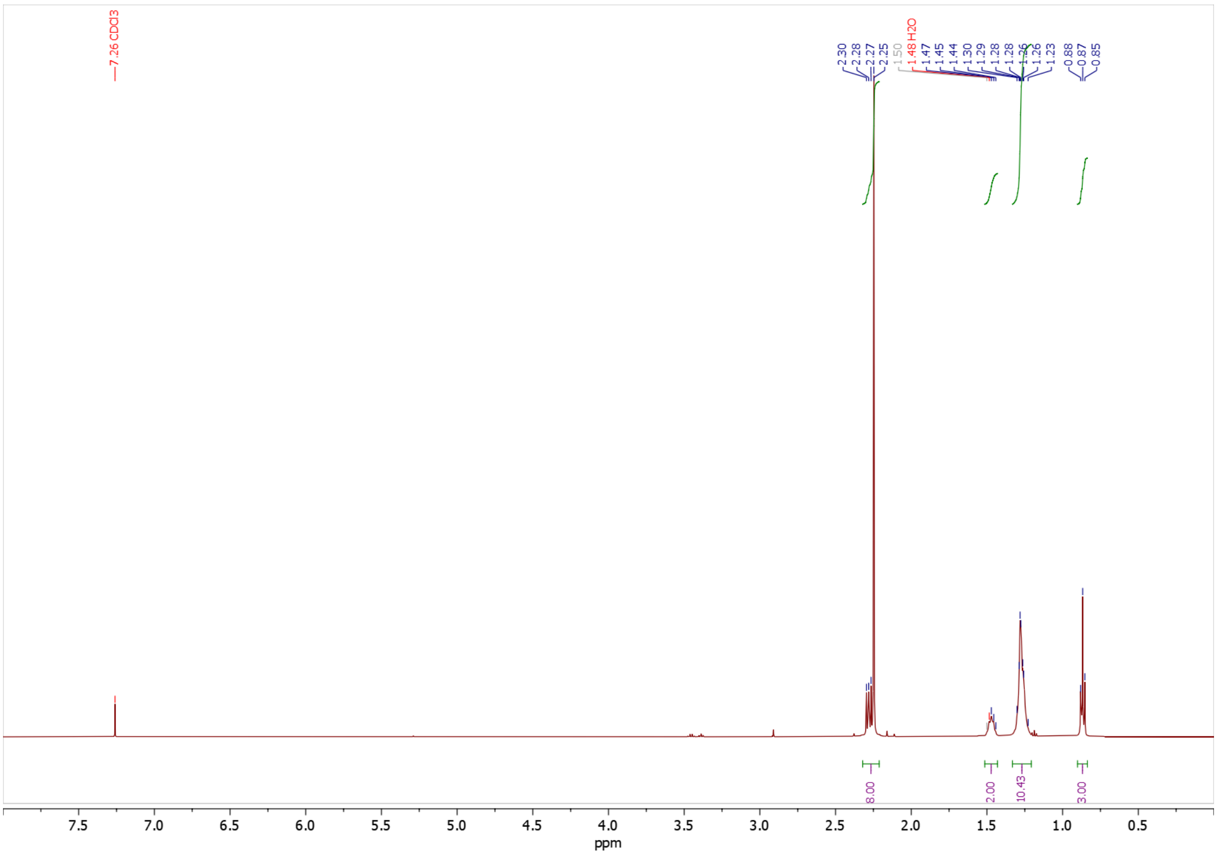

Figure S2. ^{1}H NMR spectra of *N*,*N*-dimethyloctan-1-amine

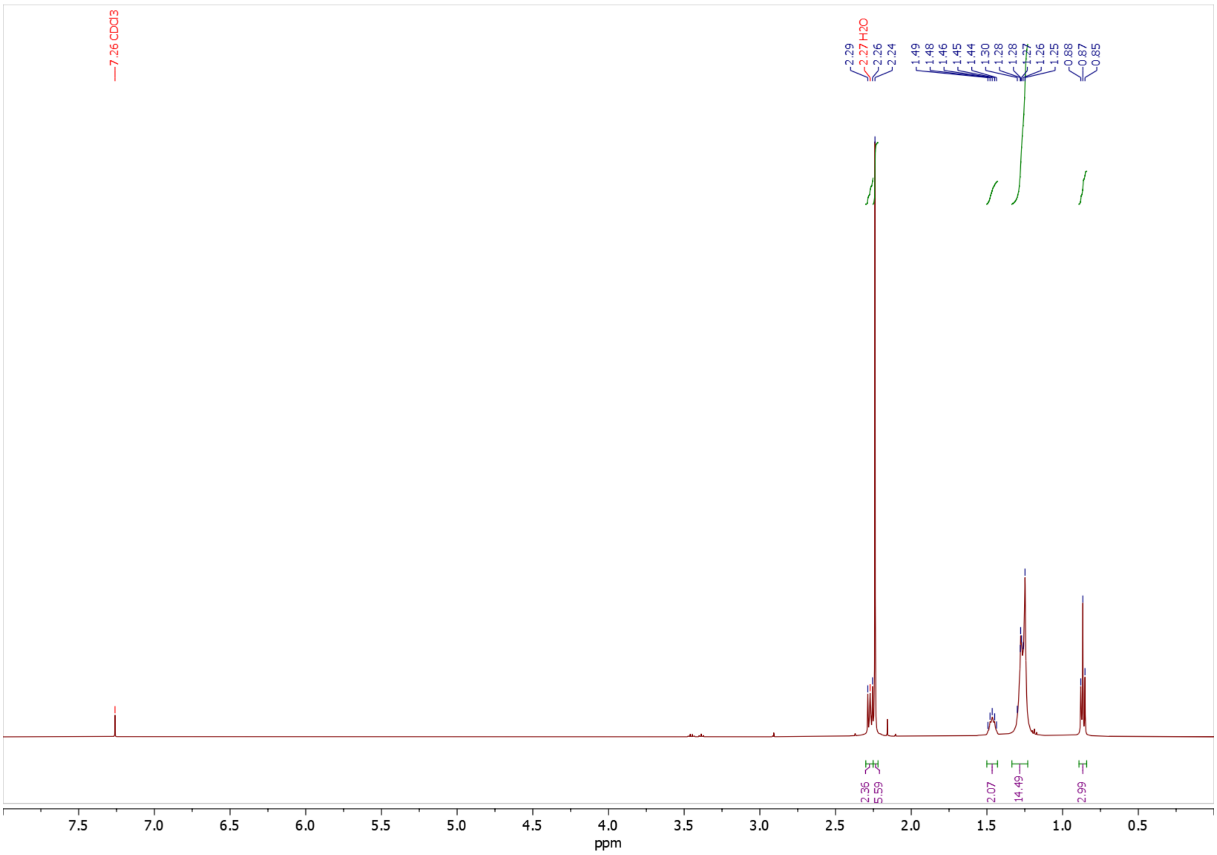

Figure S3. ^{1}H NMR spectra of *N*,*N*-dimethyldecan-1-amine

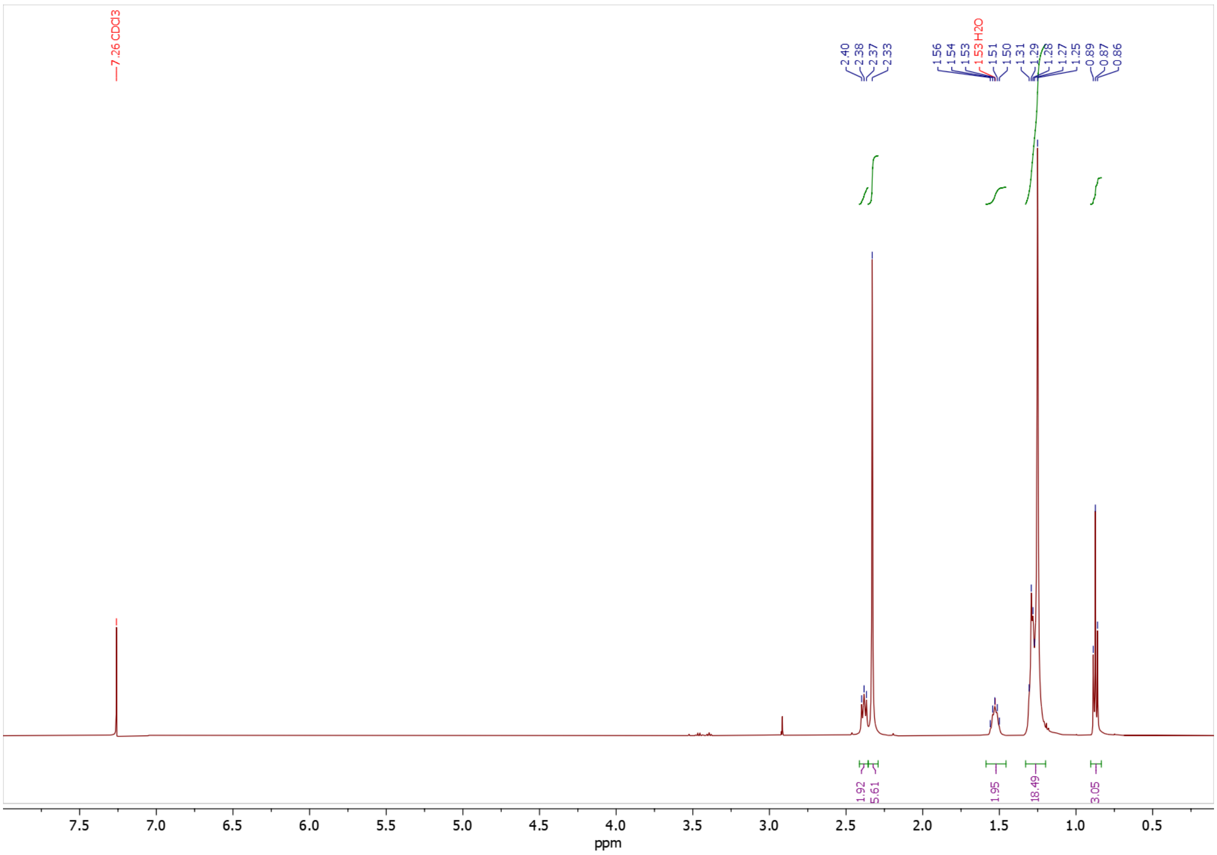

Figure S4. ^{1}H NMR spectra of *N*,*N*-dimethyldodecan-1-amine

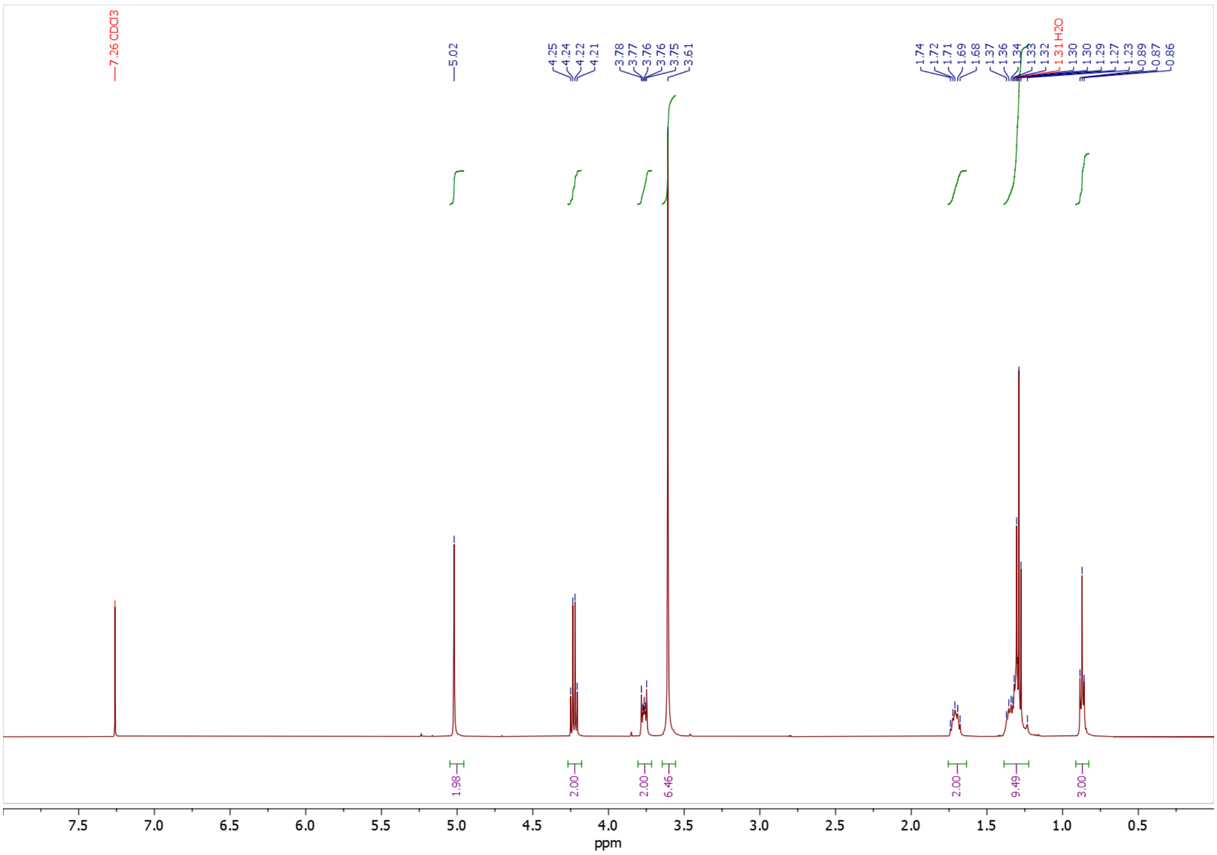

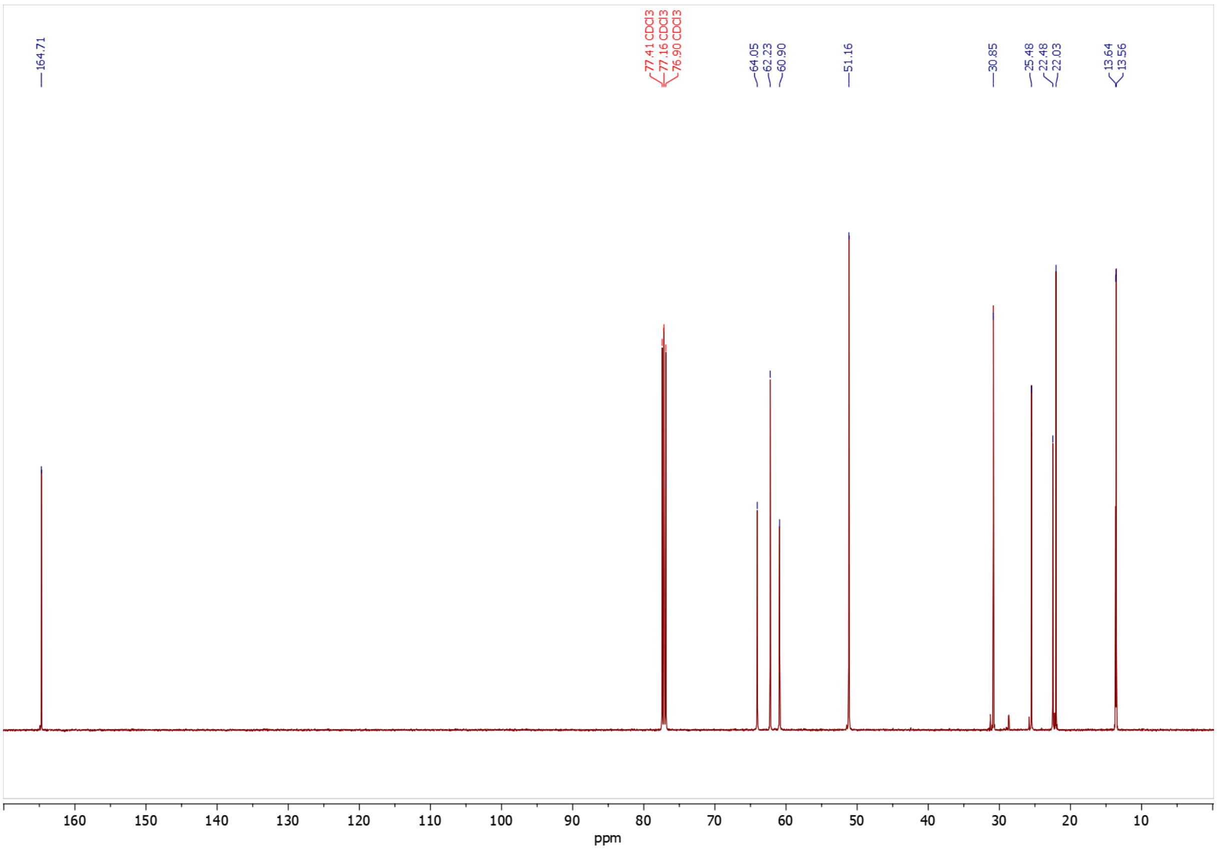

Figure S5. ^{1}H NMR and ^{13}C NMR spectra of **C_6BetC_2Cl**

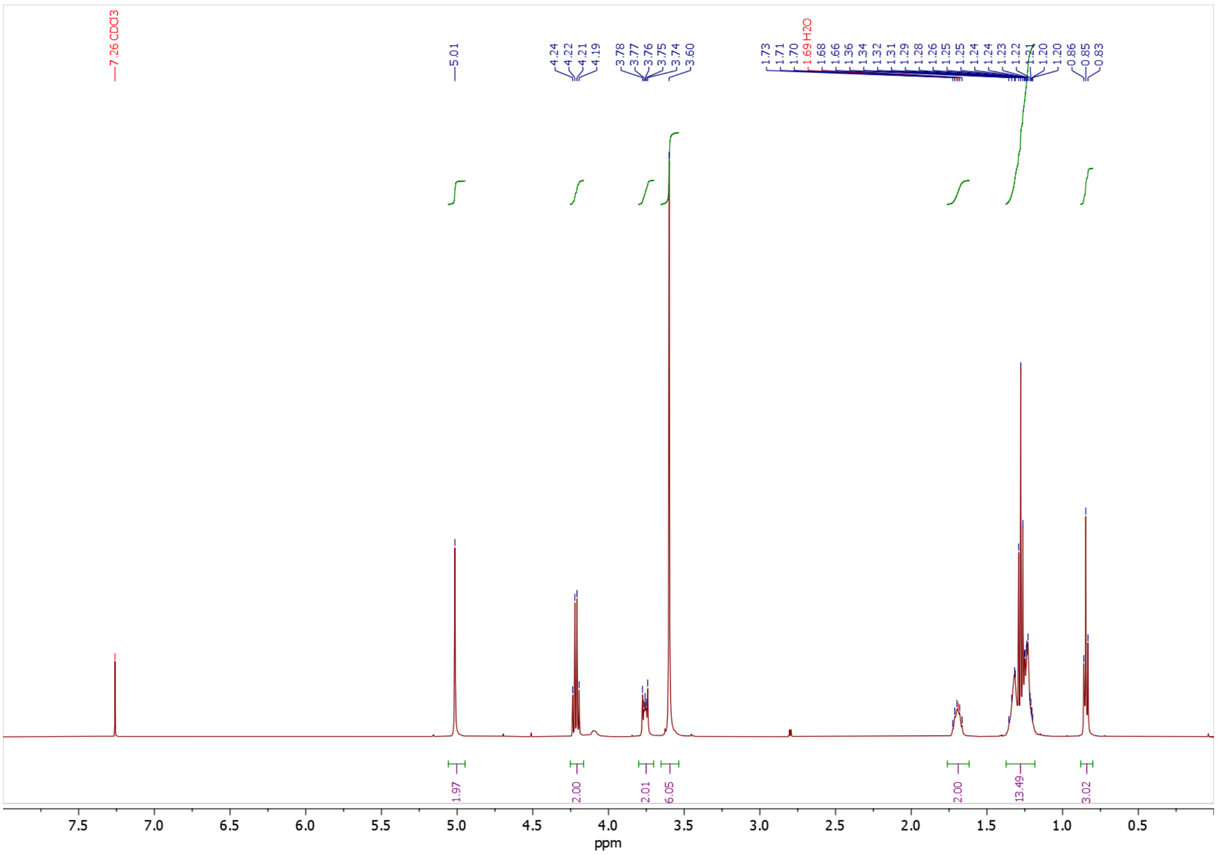

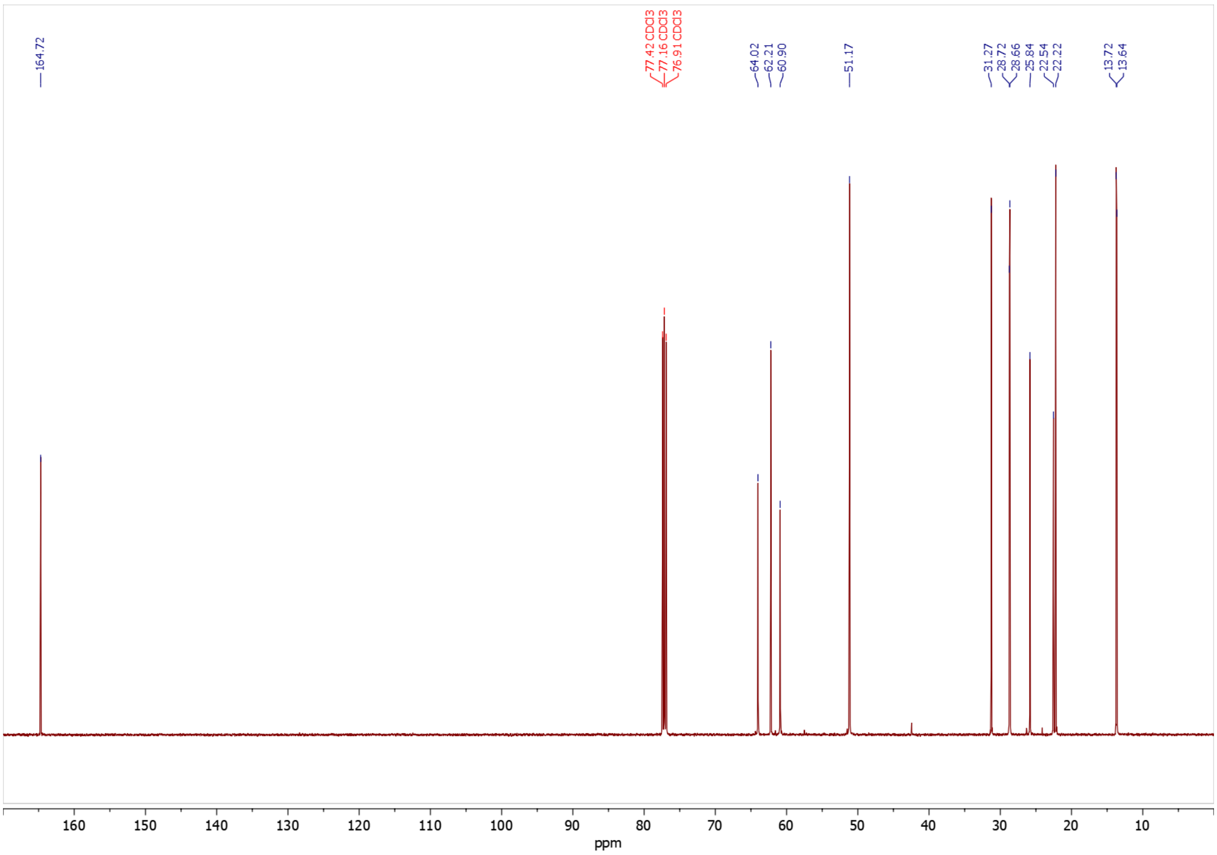

Figure S6. ^{1}H NMR and ^{13}C NMR spectra of **C_8BetC_2Cl**

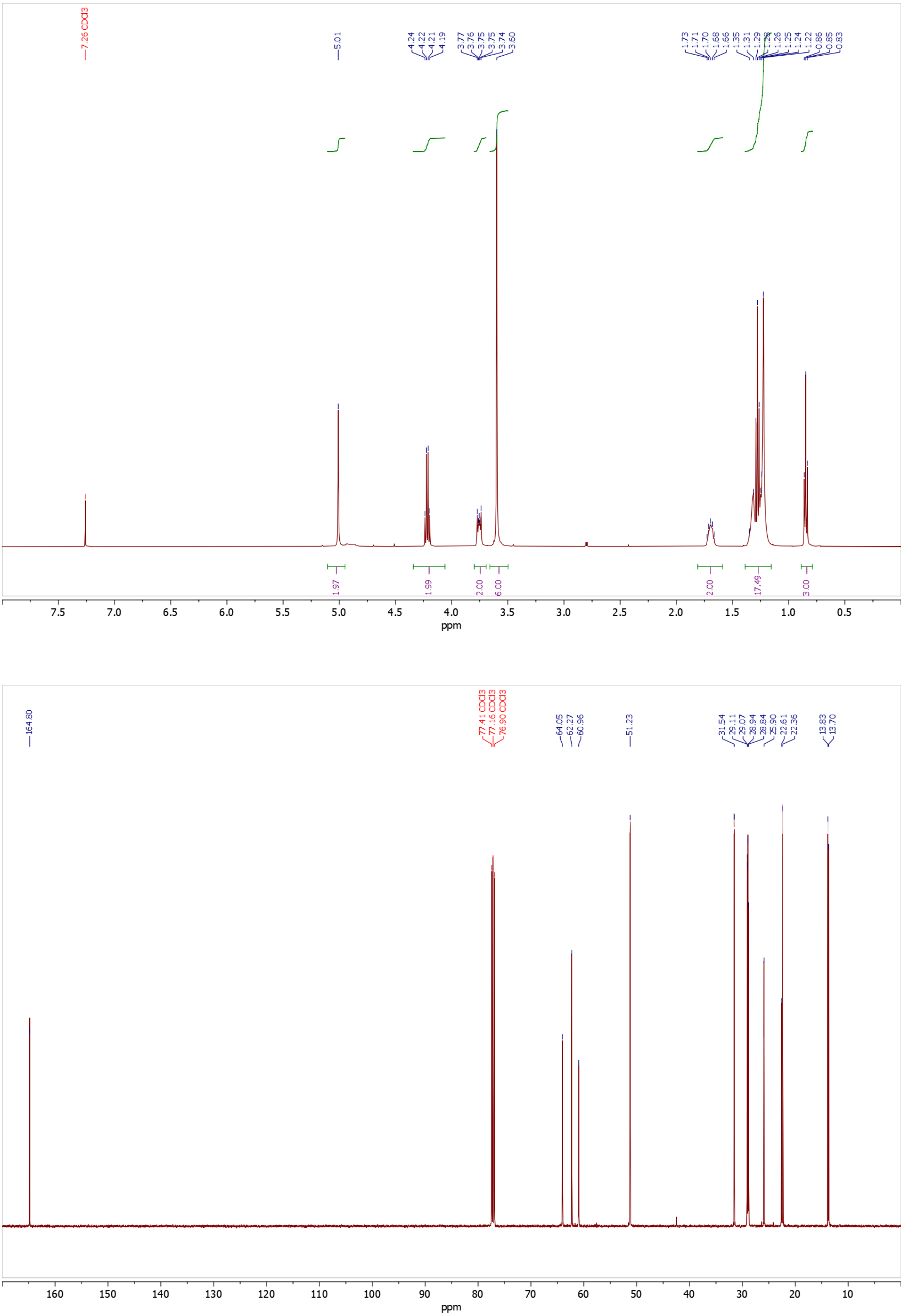

Figure S7. ^{1}H NMR and ^{13}C NMR spectra of **C_{10}BetC$_2$Cl**

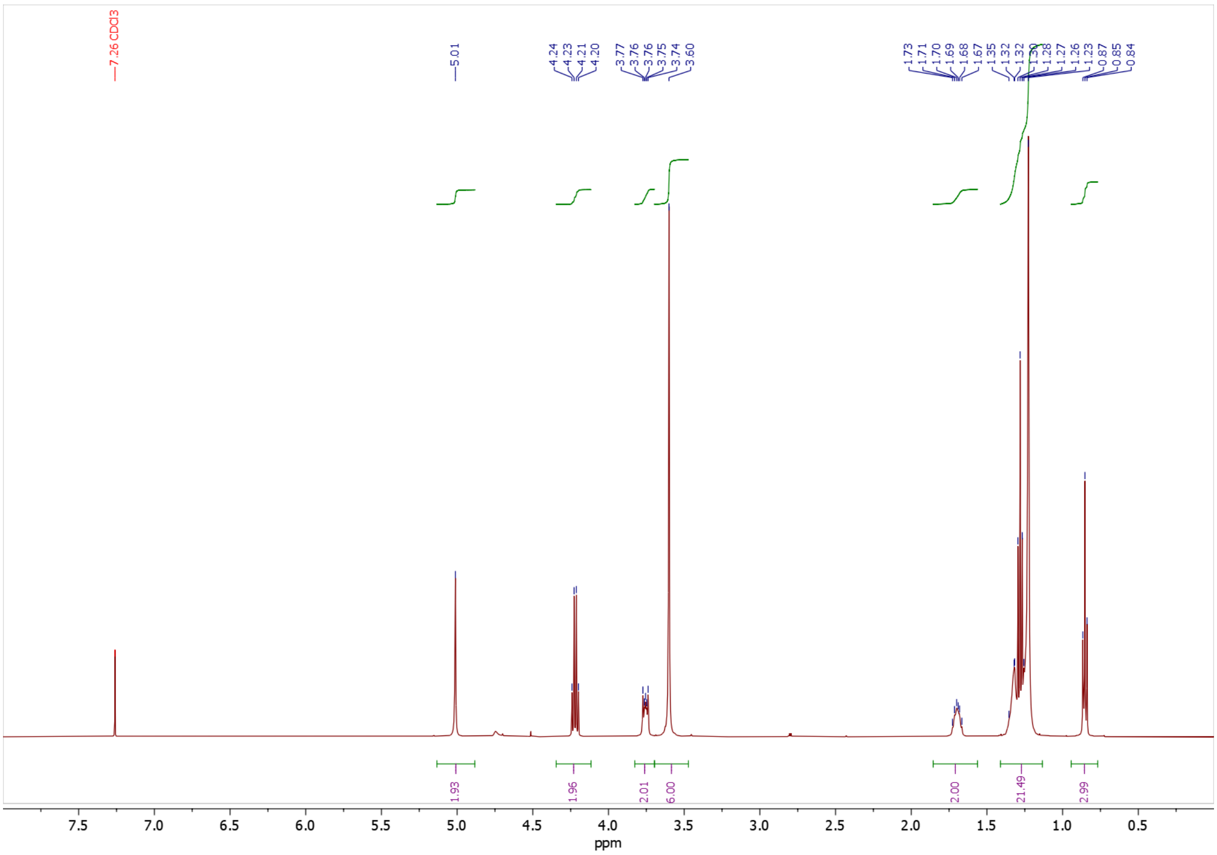

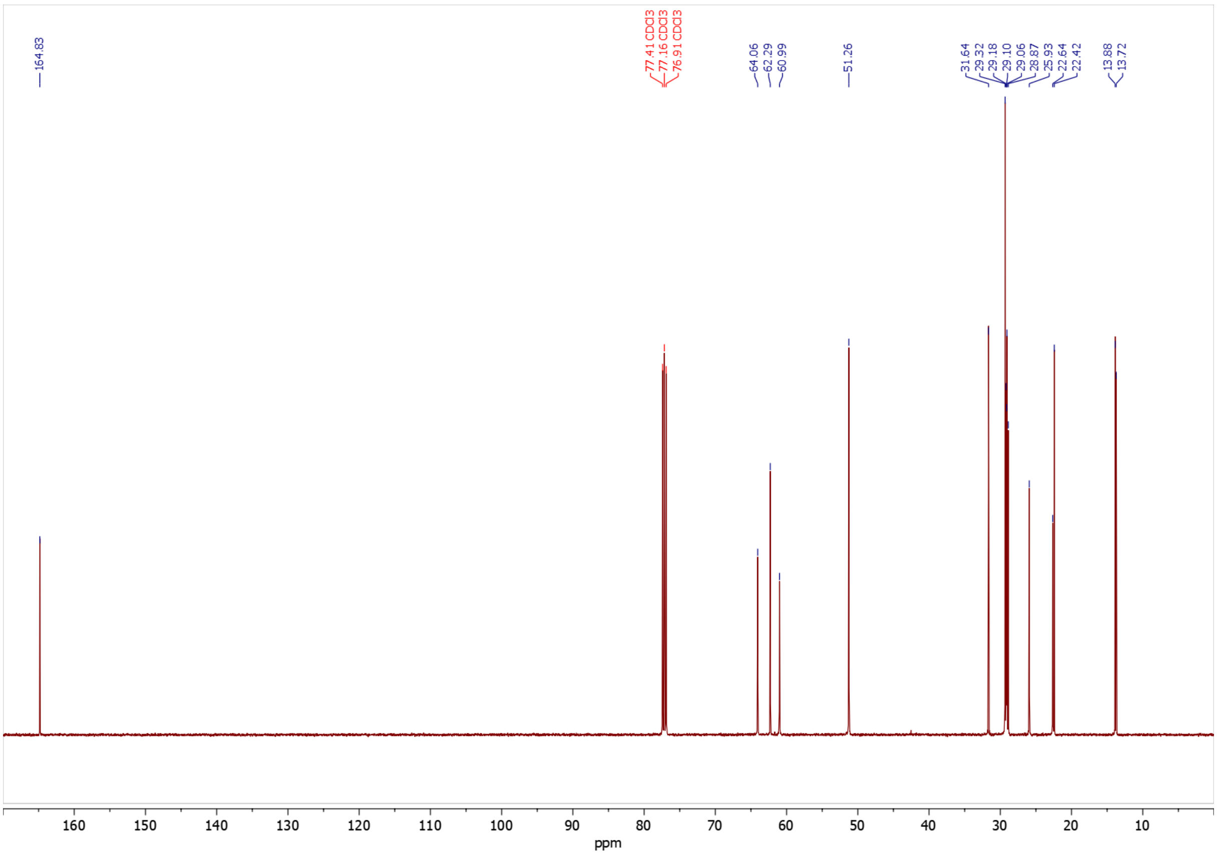

Figure S8. ^{1}H NMR and ^{13}C NMR spectra of **$C_{12}BetC_2Cl$**

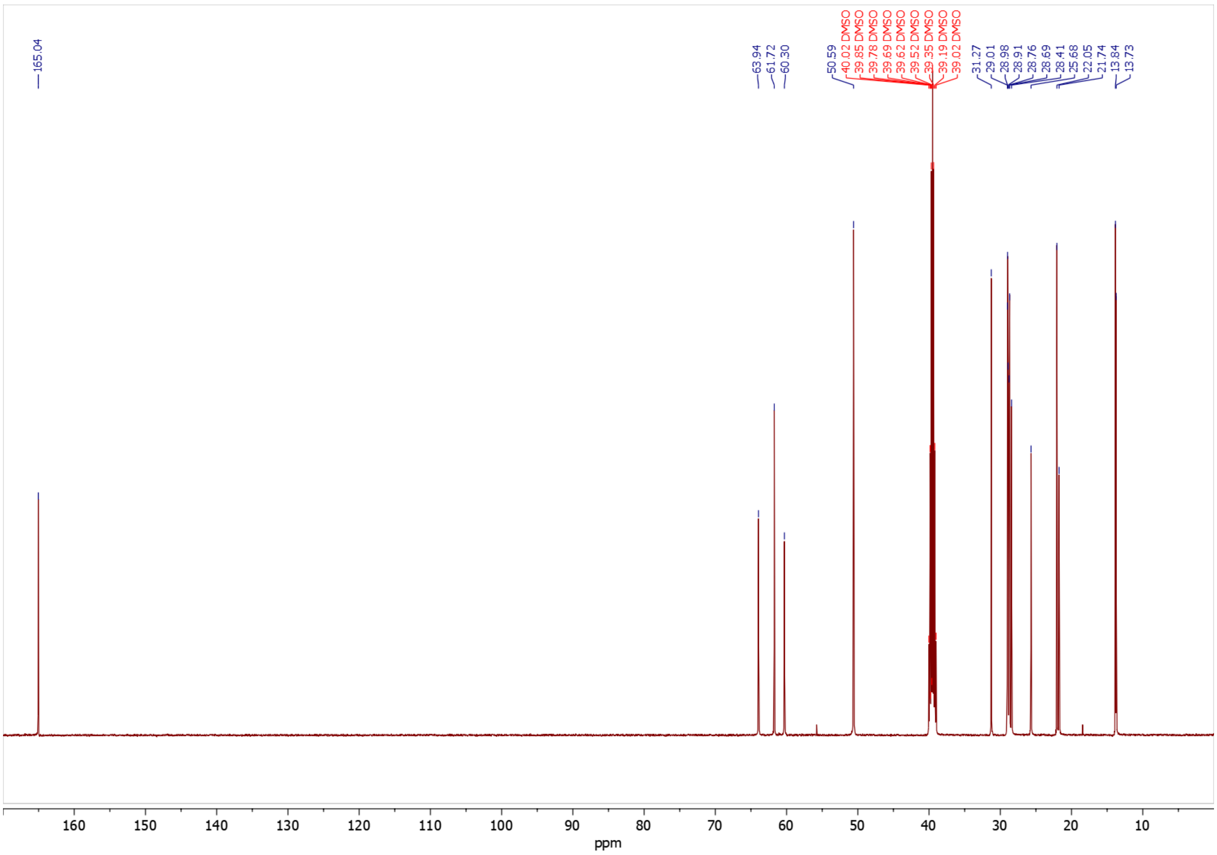

Figure S9. ^{13}C NMR spectra of **$C_{12}BetC_2Cl$** in DMSO-D6

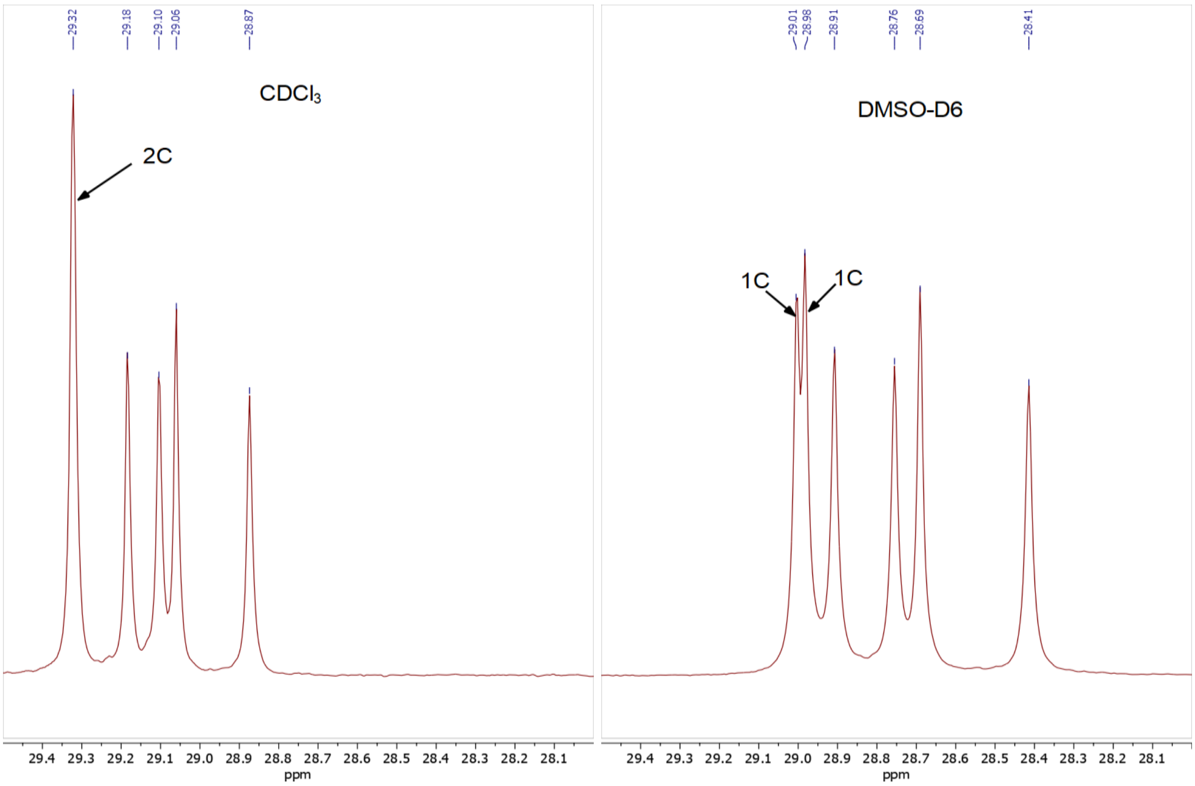

Figure S10. Comparison of ^{13}C NMR spectra of **$C_{12}BetC_2Cl$** in $CDCl_3$ and DMSO-D6

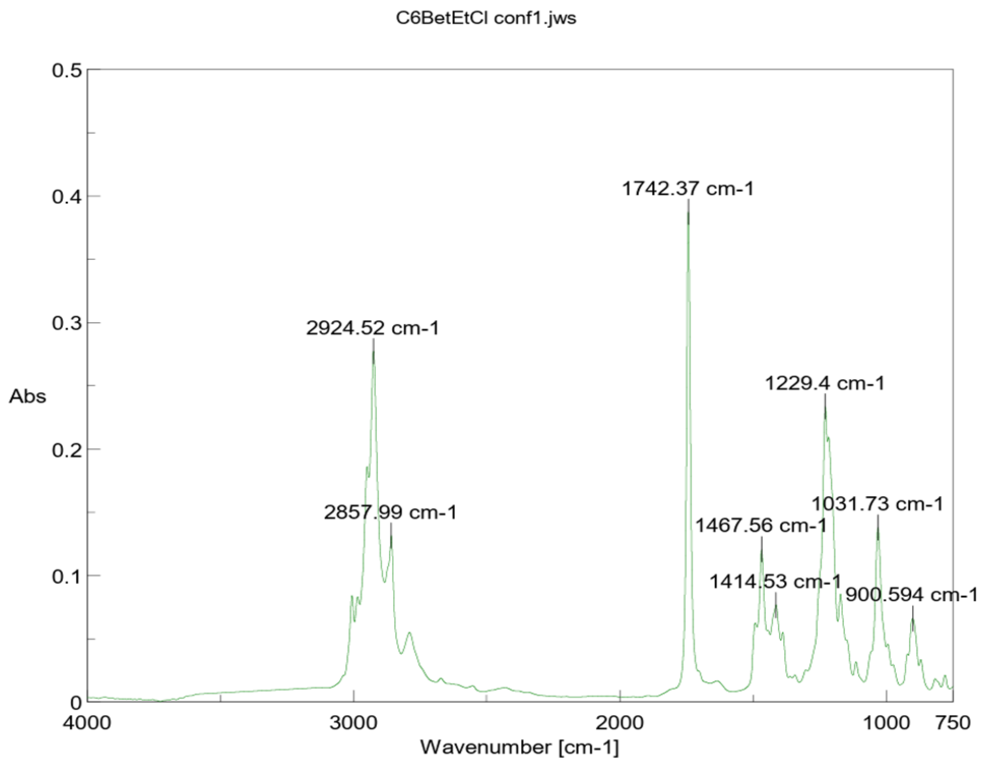

Figure S11. FTIR (ATR) spectra of **C_6BetC_2Cl**

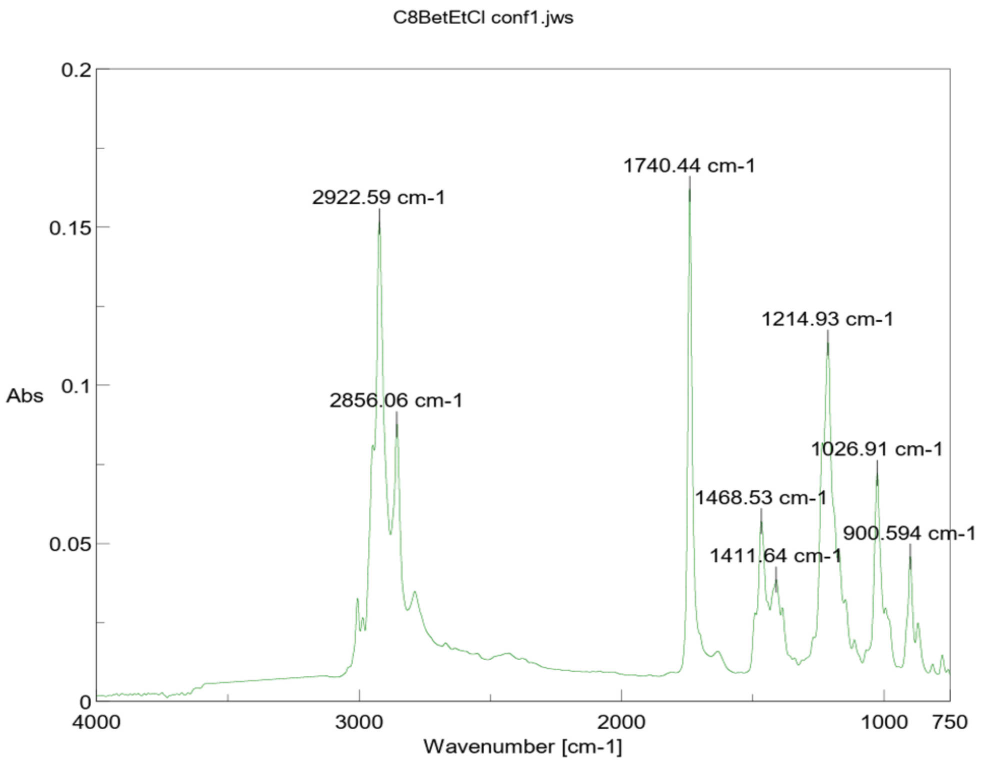

Figure S12. FTIR (ATR) spectra of **C_8BetC_2Cl**

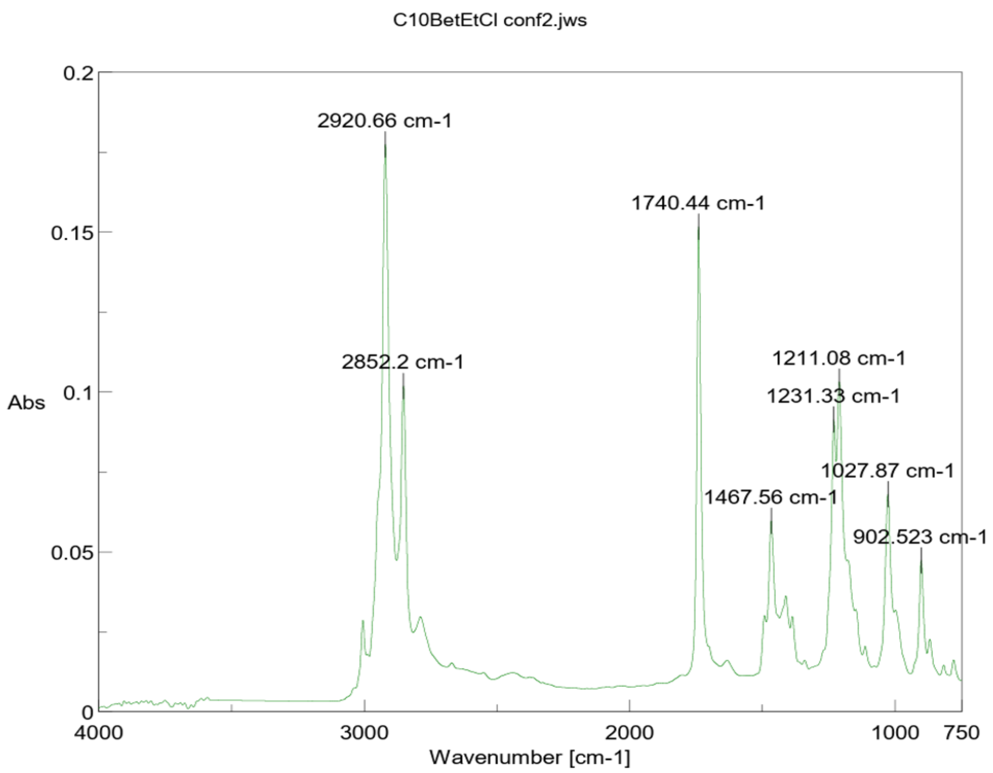

Figure S13. FTIR (ATR) spectra of **$C_{10}BetC_2Cl$**

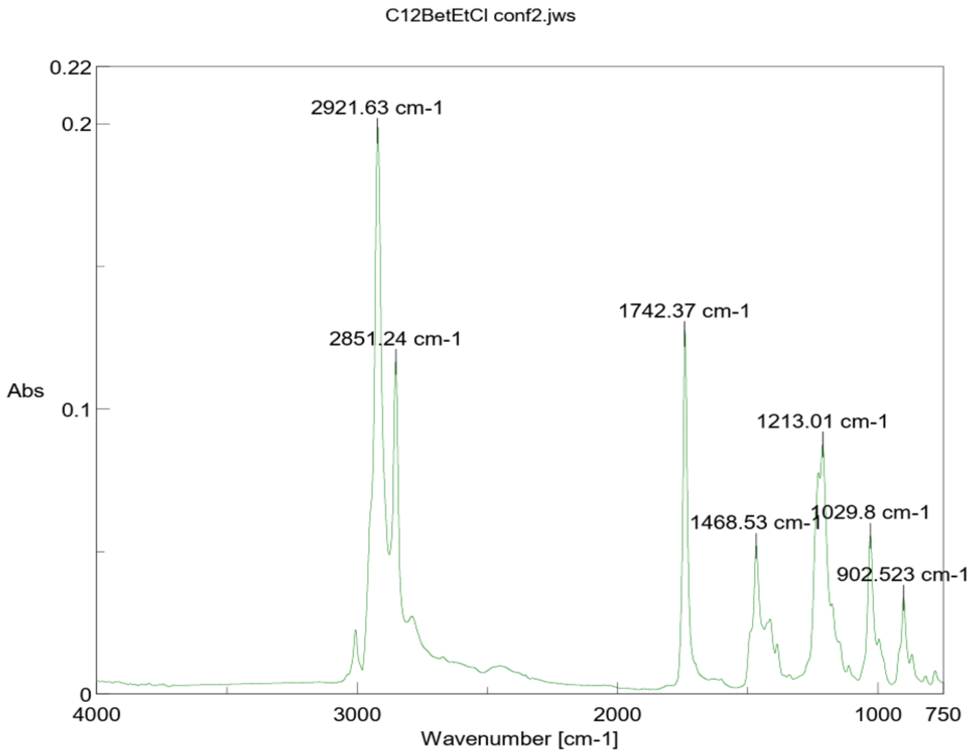

Figure S14. FTIR (ATR) spectra of **$C_{12}BetC_2Cl$**

LR-MS (m/z): calculated for [M-Cl$^-$]: 216.195, found: 216.1

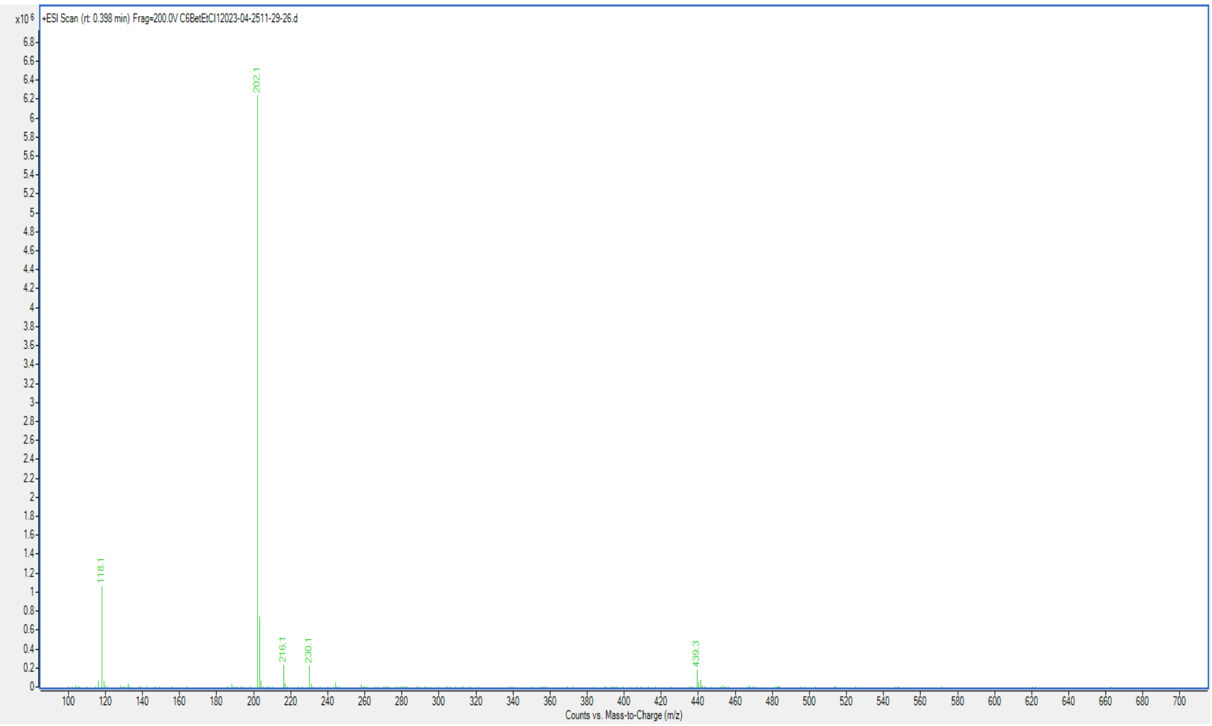

Figure S15. LR-MS (ESI) **C_6BetC_2Cl**

LR-MS (m/z): calculated for [M-Cl$^-$]: 244.227, found: 244.2

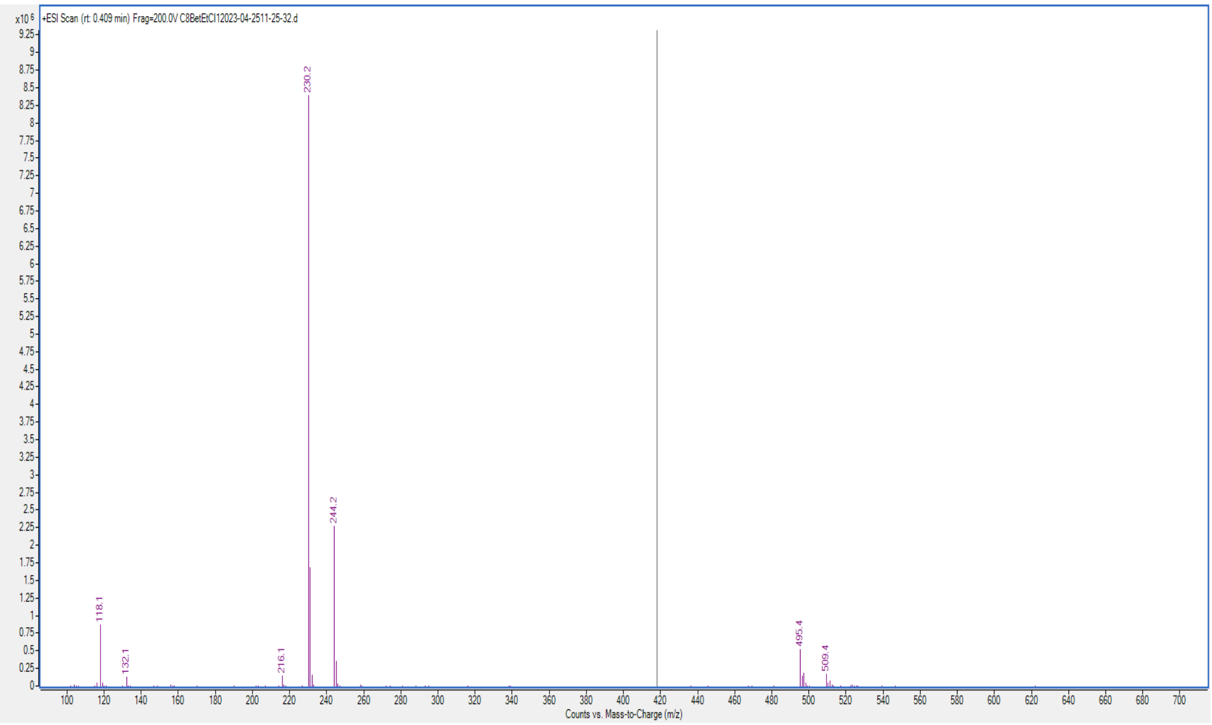

Figure S16. LR-MS (ESI) **C_8BetC_2Cl**

LR-MS (m/z): calculated for [M-Cl$^-$]: 272.258, found: 272.2

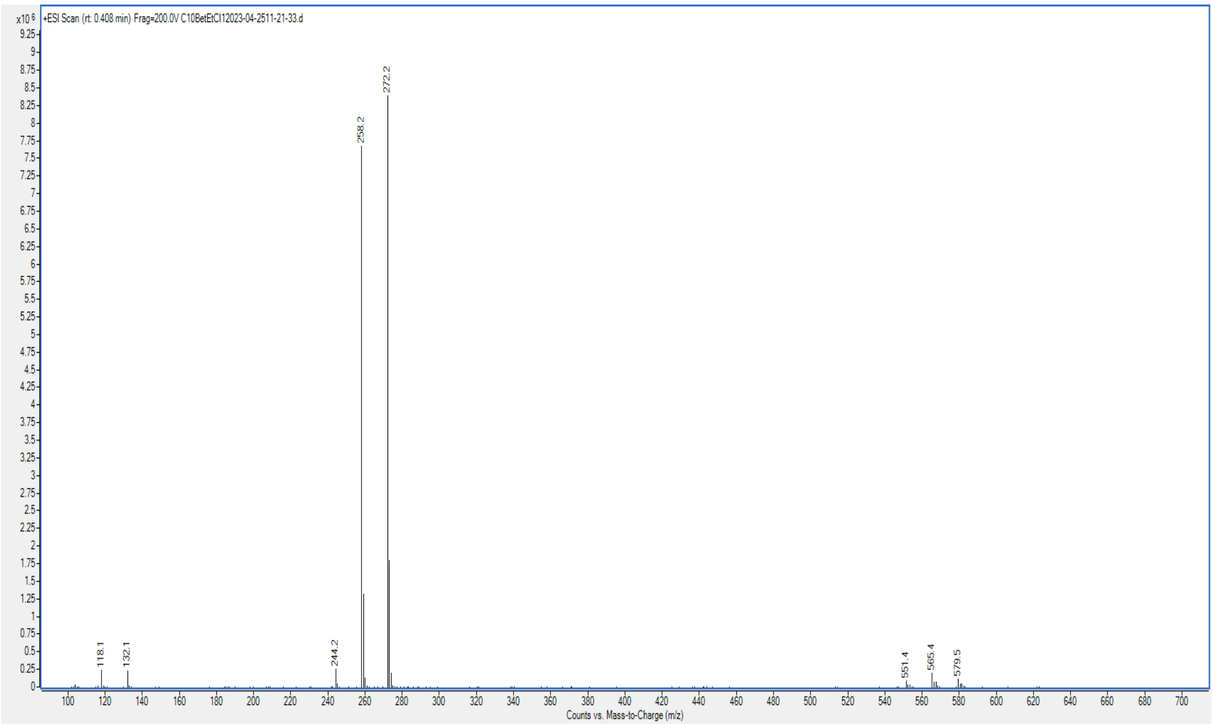

Figure S17. LR-MS (ESI) **C_{10}BetC$_2$Cl**

LR-MS (m/z): calculated for [M-Cl$^-$]: 300.289, found: 300.3

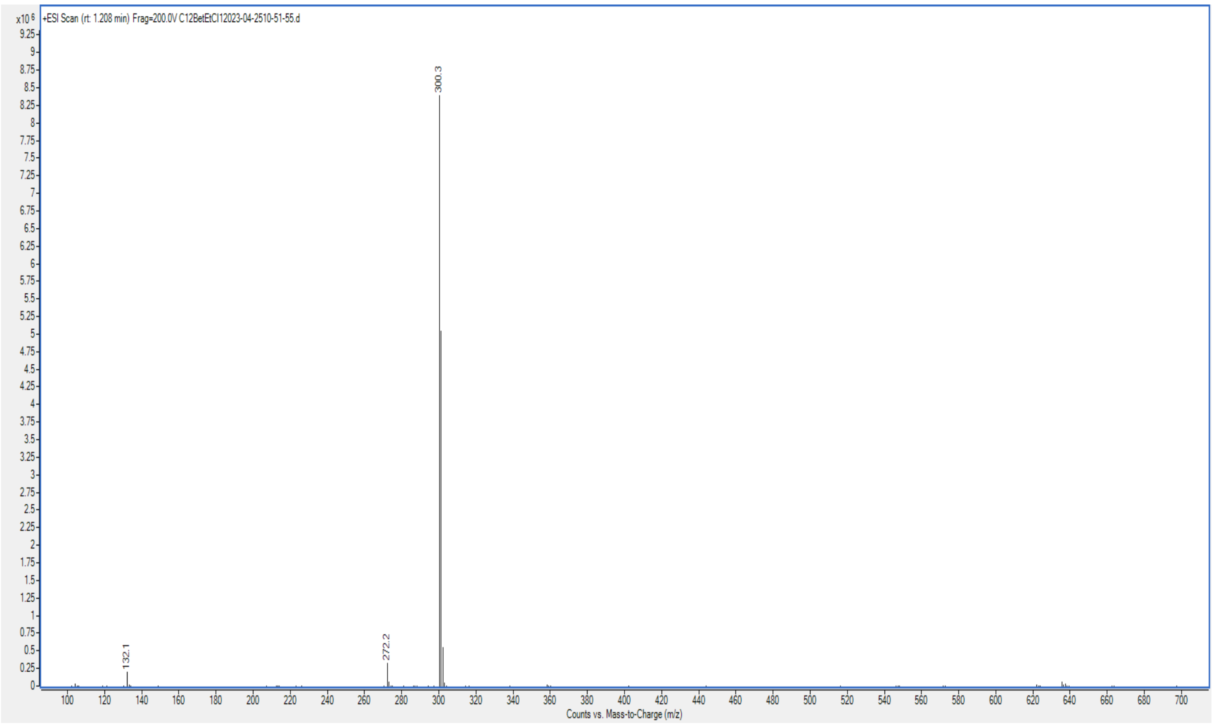

Figure S18. LR-MS (ESI) **C_{12}BetC$_2$Cl**

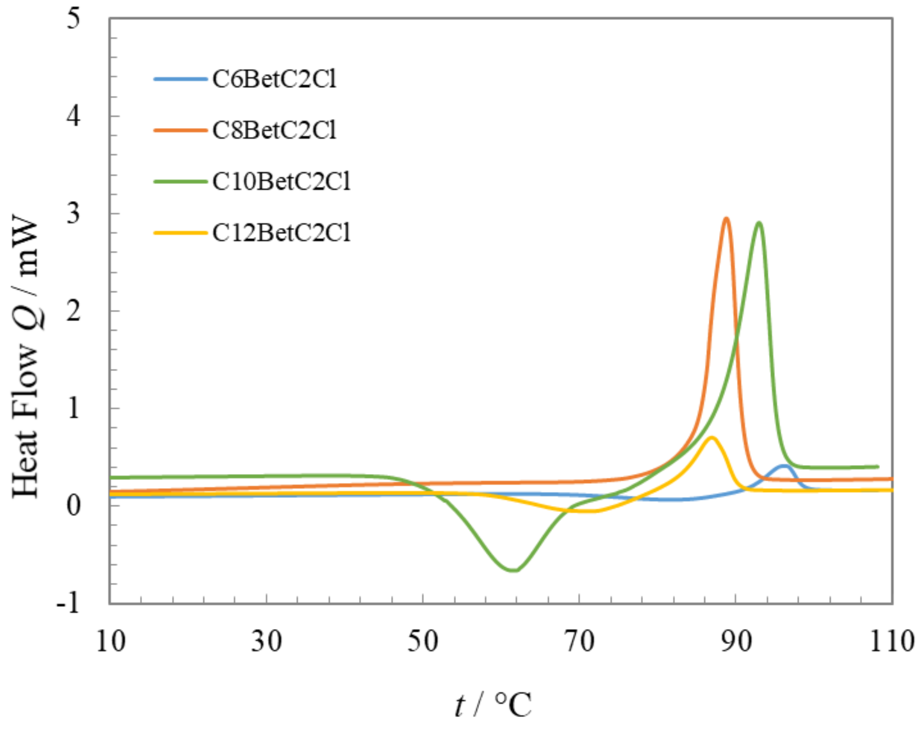

Figure S19. DSC of the synthesized *N*-alkyl betaine ethyl ester chlorides, C_nBetC_2Cl

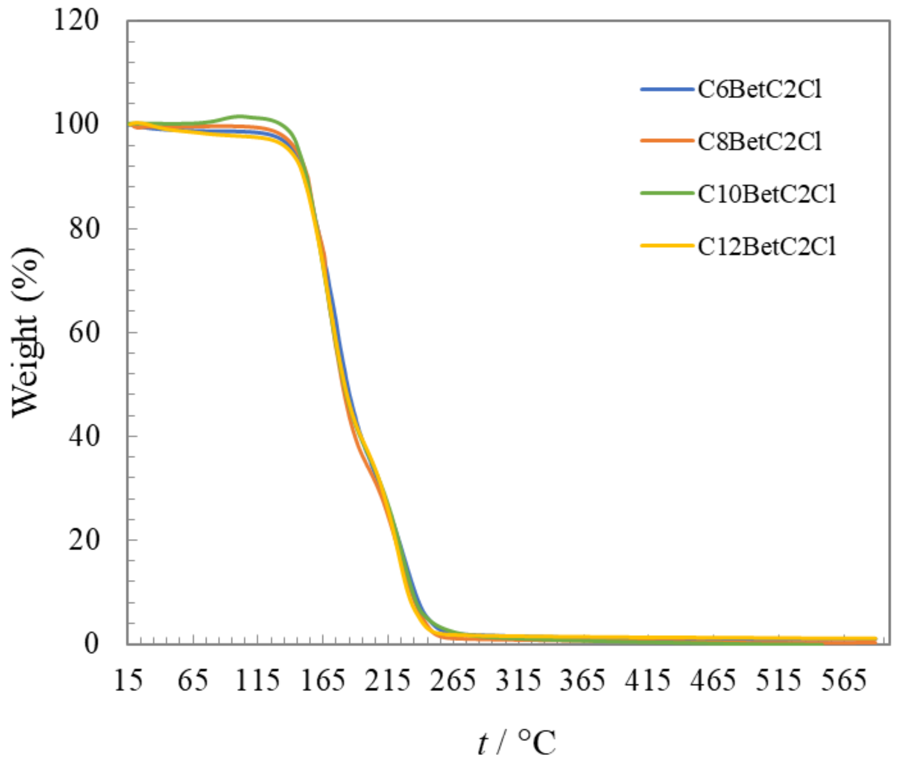

Figure S20. TGA diagrams for the synthesized *N*-alkyl betaine ethyl ester chlorides, C_nBetC_2Cl

Table S1. Characteristics of materials used for the synthesis of *N*-alkyl betaine ester chlorides in this study

Compound	CAS No.	Source	Volume percentage purity [a]
1-Bromohexane	111-25-1	Aldrich	98
1-Bromooctane	111-83-1	Aldrich	99
1-Bromodecane	112-29-8	Aldrich	98
1-Bromododecane	143-15-7	Aldrich	95
Dimethylamine hydrochloride	506-59-2	Acros Organics (Belgium)	99
Diethyl ether	60-29-7	POCH S.A. (Poland)	99.5
Ethanol	64-17-5	POCH S.A. (Poland)	99.8 [b]
Acetone	67-64-1	POCH S.A. (Poland)	99.5
Sodium hydroxide	1310-73-2	POCH S.A. (Poland)	99
Ethyl chloroacetate	105-39-5	POCH S.A. (Poland)	99

[a] according to supplier; [b] mass percentage

Table S2. Density, speed of sound, surface tension and dynamic viscosity of aqueous solutions of *N*-alkyl betaine ethyl esters chlorides, C_nBetC_2Cl (for n = 6, 8, 10 and 12 (1)) at 25 °C, and for $C_{12}BetC_2Cl$ at temperatures t = (15 – 45) °C, with a step of 10 °C

m / (mol·kg^{-1})	ρ / (g·cm^{-3})	c / (m·s^{-1})	γ / (mN·m^{-1})	η / (mPa·s)
		C_6BetC_2Cl at t = 25 °C		
0	0.997043	1496.71	71.99	0.890
0.00020	0.997047	1496.73	70.79	0.888
0.00058	0.997053	1496.80	70.21	0.893
0.00095	0.997059	1496.87	69.01	0.896
0.00232	0.997077	1497.12	68.65	0.898
0.00488	0.997114	1497.49	67.50	0.899
0.01103	0.997198	1498.66	66.16	0.902
0.02046	0.997330	1500.69	64.06	0.908
0.05421	0.997792	1507.10	59.49	0.924
0.07805	0.998123	1511.58	57.40	0.943
0.10062	0.998440	1517.03	55.36	0.960
0.16739	0.999358	1527.75	52.39	1.010
0.23096	1.000238	1538.83	50.64	1.050
		C_8BetC_2Cl at t = 25 °C		
0.00010	0.997044	1496.75	70.42	0.890
0.00020	0.997046	1496.77	68.27	0.890
0.00050	0.997052	1496.85	68.11	0.898
0.00098	0.997056	1496.98	67.47	0.900

0.00194	0.997064	1497.25	63.90	0.894
0.00504	0.997101	1497.94	59.02	0.904
0.01006	0.997154	1499.04	57.74	0.898
0.02005	0.997258	1501.18	55.21	0.904
0.05038	0.997587	1507.94	50.35	0.928
0.07673	0.997874	1513.15	46.89	-
0.10098	0.998148	1518.86	43.93	0.984
0.12353	0.998371	1522.49	41.89	-
0.14823	0.998601	1527.65	40.08	1.018
0.17219	0.998883	1531.74	39.74	-
0.20934	0.999171	1537.09	38.95	1.066
0.29177	0.999941	1548.02	38.53	-
0.35362	1.000260	1552.39	37.83	1.208

$C_{10}BetC_2Cl$ at t = 25 °C

0.00008	0.997044	1496.73	70.49	0.896
0.00022	0.997045	1496.76	69.86	0.899
0.00053	0.997047	1496.84	69.47	0.902
0.00094	0.997050	1496.95	68.23	0.898
0.00199	0.997058	1497.22	65.24	0.900
0.00528	0.997081	1498.05	58.91	0.896
0.00991	0.997114	1499.20	53.67	0.904
0.01964	0.997182	1501.57	49.01	0.911
0.04802	0.997373	1508.17	39.57	0.939
0.07519	0.997424	1511.39	38.82	0.969
0.10186	0.997397	1512.72	38.73	1.003
0.14891	0.997336	1514.59	37.69	1.068
0.18708	0.997288	1516.09	37.79	1.126
0.31366	0.997156	1521.15	37.22	1.336

$C_{12}BetC_2Cl$ at t = 25 °C (1)

0.00012	0.997043	1496.81	67.47	0.889
0.00030	0.997045	1496.85	66.30	0.896
0.00077	0.997047	1496.97	64.14	0.895
0.00101	0.997049	1497.04	61.79	0.896
0.00222	0.997051	1497.35	56.13	0.892
0.00547	0.997063	1498.26	47.53	0.896
0.01033	0.99708	1499.55	39.80	0.902
0.01832	0.997054	1500.48	38.89	0.913
0.04753	0.996863	1501.53	38.49	0.969
0.07313	0.996701	1502.61	38.53	1.020
0.09783	0.996551	1503.52	37.97	1.068
0.17110	0.996132	1506.53	37.24	1.218
0.25323	0.995686	1509.87	37.21	1.434

$C_{12}BetC_2Cl$ at t = 15 °C

0	0.999089	1466.31	72.39	1.138
0.00007	0.999097	1466.36	71.11	1.137
0.00022	0.999100	1466.43	69.00	1.139
0.00045	0.999104	1466.53	66.71	1.138
0.00100	0.999107	1466.67	60.41	1.140
0.00241	0.999118	1467.16	55.84	1.142
0.00483	0.999135	1467.94	48.63	1.146

0.01012	0.999172	1469.64	42.68	1.156
0.01992	0.999172	1471.22	40.07	1.174
0.05141	0.999038	1472.96	39.99	1.263
0.07442	0.998946	1474.32	39.57	1.317
0.09959	0.998850	1475.85	39.40	1.388
0.17494	0.998574	1480.54	38.97	1.599
0.20120	0.998487	1482.17	38.66	1.664
0.29168	0.998192	1487.71	38.06	1.968
$C_{12}BetC_2Cl$ at t = 25 °C (2)				
0	0.997043	1496.73	71.99	0.890
0.00007	0.997053	1496.66	70.92	0.889
0.00022	0.997055	1496.70	68.47	0.891
0.00045	0.997058	1496.79	66.06	0.890
0.00100	0.997059	1496.91	58.80	0.892
0.00241	0.997066	1497.34	54.02	0.894
0.00483	0.997075	1497.98	47.54	0.896
0.01012	0.997096	1499.42	39.49	0.903
0.01992	0.997056	1500.44	38.94	0.918
0.05141	0.99685	1501.57	38.61	0.990
0.07442	0.996705	1502.46	37.71	1.030
0.09959	0.996555	1503.48	37.40	1.086
0.17494	0.996114	1506.58	37.31	1.247
0.20120	0.995971	1507.64	37.17	1.301
0.29168	0.995494	1511.24	37.23	1.501
$C_{12}BetC_2Cl$ at t = 35 °C				
0	0.994054	1519.25	70.60	0.719
0.00007	0.994052	1519.69	68.12	0.718
0.00022	0.994052	1519.72	67.31	0.720
0.00045	0.994053	1519.79	62.67	0.718
0.00100	0.994052	1519.92	56.80	0.721
0.00241	0.994054	1520.26	52.99	0.721
0.00483	0.994056	1520.80	46.61	0.722
0.01012	0.994057	1521.98	39.05	0.728
0.01992	0.993995	1522.68	38.27	0.741
0.05141	0.993731	1523.19	37.85	0.797
0.07442	0.993544	1523.61	37.16	0.830
0.09959	0.993347	1524.10	36.90	0.875
0.17494	0.992779	1525.64	36.62	1.003
0.20120	0.992589	1526.15	36.70	1.044
0.29168	0.991966	1527.93	36.66	1.209
$C_{12}BetC_2Cl$ at t = 45 °C				
0	0.990235	1536.62	69.06	0.598
0.00007	0.990240	1536.66	65.99	0.597
0.00022	0.990235	1536.68	64.97	0.599
0.00045	0.990241	1536.73	60.75	0.598
0.00100	0.990240	1536.84	55.27	0.599
0.00241	0.990232	1537.11	52.09	0.599
0.00483	0.990228	1537.54	45.29	0.600
0.01012	0.990219	1538.51	38.60	0.605
0.01992	0.990142	1539.03	37.75	0.615

0.05141	0.989830	1538.91	37.32	0.660
0.07442	0.989609	1538.88	36.71	0.687
0.09959	0.989376	1538.86	36.31	0.723
0.17494	0.988700	1538.89	36.08	0.828
0.20120	0.988476	1538.89	36.00	0.862
0.29168	0.987733	1539.02	36.10	0.997

Standard uncertainties: $u(t)$ = 0.01 °C, for density and speed of sound; $u(t)$ = 0.02 °C for viscosity; $u(t)$ = 0.1 °C for surface tension; $u(P)$= 0.01 MPa, $u(m) = 1\cdot10^{-5}$ mol·kg^{-1}, $u(\rho) = 2\cdot10^{-5}$ g·cm^{-3}, $u(c)$ = 0.1 m·s^{-1}; $u(\gamma)$ = 0.2 mN·m^{-1}; $u(\eta)$ = 0.5%

Table S3. Coefficients of equations: $y = \sum_{i=0}^{n=2} y_i \cdot m^i$ describing concentration dependence of density, ρ, and speed of sound, c, and: $\gamma = a + b \cdot \log\, m$ for surface tension, γ, of aqueous solutions of *N*-alkyl betaine ethyl esters chlorides, C_nBetC$_2$Cl (for n = 6, 8, 10, 12 (1)) at 25 °C, and for C$_{12}$BetC$_2$Cl at temperatures t = (15 - 45) °C, with a step of 10 K, together with the mean deviations from the regression line: $\delta\rho$, δc, $\delta\gamma$ and CMC (if attainable) calculated based on the intersection of the curves before and after CMC; for density and speed of sound there is one equation for C$_6$BetC$_2$Cl and C$_8$BetC$_2$Cl, and two independent equations were found, before and after CMC for C$_{10}$BetC$_2$Cl and C$_{12}$BetC$_2$Cl.

C$_6$BetC$_2$Cl at t = 25 °C			
ρ_0 / (g·cm^{-3})	ρ_1 / (g·cm^{-3}·kg·mol^{-1})	ρ_2 / (g·cm^{-3}·kg^2·mol^{-2})	$\delta\rho$
0.9970450 ± 0.0000008	0.013826 ± 0.000026	-0.0000120 ±0.000124	0.000002
c_0 / (m·s^{-1})	c_1 / (m·s^{-1}·kg·mol^{-1})	c_2 / (m·s^{-1}·kg^2·mol^{-2})	δc
1496.62 ± 0.13	203.5 ± 4.4	-91 ± 21	0.3
C$_8$BetC$_2$Cl at t = 25 °C (for density and speed of sound)			
ρ_0 / (g· cm^{-3})	ρ_1 / (g·cm^{-3}· kg·mol^{-1})	ρ_2 / (g·cm^{-3}· kg^2·mol^{-2})	$\delta\rho$
0.9970370 ± 0.0000090	0.01175 ± 0.00019	-0.00708 ± 0.00060	0.00003
c_0 / (m·s^{-1})	c_1 / (m·s^{-1}·kg·mol^{-1})	c_2 / (m·s^{-1}·kg^2·mol^{-2})	δc
1496.62 ± 0.12	242.6 ± 2.4	-236.0 ± 7.7	0.4
C$_8$BetC$_2$Cl at t = 25 °C (surface tension below CMC)			
a	b	$\delta\gamma$	
22.24 ± 1.10	-21.80 ± 1.01	0.3	
C$_8$BetC$_2$Cl at t = 25 °C (surface tension after CMC)			
a	b	$\delta\gamma$	
35.31 ± 0.37	-5.71 ± 0.56	0.2	
***CMC* / mol·kg^{-1}**	**0.154**		
C$_{10}$BetC$_2$Cl at t = 25 °C (below CMC)			
ρ_0 / (g·cm^{-3})	ρ_1 / (g·cm^{-3}·kg·mol^{-1})	ρ_2 / (g·cm^{-3}·kg^2·mol^{-2})	$\delta\rho$

0.9970441 ± 0.0000006	0.006886 ± 0.000036	-	0.000002
c_0 / (m·s^{-1})	c_1 / (m·s^{-1}·kg·mol^{-1})	c_2 / (m·s^{-1}·kg^{2}·mol^{-2})	δc
1496.75 ± 0.02	238.2 ± 1.4	-	0.06
a	*b*	$\delta\gamma$	
13.96 ± 2.43	-19.89 ±1.31	0.9	
$C_{10}BetC_2Cl$ at *t* = 25 °C (after CMC)			
ρ_0 / (g·cm^{-3})	ρ_1 / (g·cm^{-3}·kg·mol^{-1})	ρ_2 / (g·cm^{-3}·kg^{2}·mol^{-2})	$\delta\rho$
0.997508 ± 0.000006	-0.001135 ± 0.000031	-	0.00001
***CMC* (ρ) / mol·kg^{-1}**	**0.058**		
c_0 / (m·s^{-1})	c_1 / (m·s^{-1}·kg·mol^{-1})	c_2 / (m·s^{-1}·kg^{2}·mol^{-2})	δc
1508.49 ± 0.11	40.48 ± 0.61	-	0.11
***CMC* (*c*) / mol·kg^{-1}**	**0.059**		
a	*b*	$\delta\gamma$	
35.74 ± 0.45	-2.77 ± 0.52	0.25	
***CMC* (γ) / mol·kg^{-1}**	**0.053**		
$C_{12}BetC_2Cl$ at *t* = 25 °C (below CMC) (1)			
ρ_0 / (g·cm^{-3})	ρ_1 / (g·cm^{-3}·kg·mol^{-1})	ρ_2 / (g·cm^{-3}·kg^{2}·mol^{-2})	$\delta\rho$
0.9970437 ± 0.0000004	0.00352 ± 0.00010	-	0.0000001
c_0 / (m·s^{-1})	c_1 / (m·s^{-1}·kg·mol^{-1})	c_2 / (m·s^{-1}·kg^{2}·mol^{-2})	δc
1496.76 ± 0.01	270.6 ± 1.8	-	0.02
a	*b*	$\delta\gamma$	
-1.03 ± 3.05	-21.10 ± 1.16	1.1	
$C_{12}BetC_2Cl$ at *t* = 25 °C (after CMC) (1)			
ρ_0 / (g·cm^{-3}·kg·mol^{-1})	ρ_1 / (g·cm ·kg·mol$^{3-1}$)	ρ_2 / (g·cm^{-3}·kg·mol^{-1})	$\delta\rho$
0.997136 ± 0.000013	-0.00579 ± 0.0001	-	0.00002
***CMC* (ρ) / mol·kg^{-1}**	**0.01**		
c_0 / (m·s^{-1})	c_1 / (m·s^{-1}·kg·mol^{-1})	c_2 / (m·s^{-1}·kg^{2}·mol^{-2})	δc
1499.66 ± 0.04	40.17 ± 0.32	-	0.06
***CMC* (*c*) / mol·kg^{-1}**	**0.013**		
a	*b*	$\delta\gamma$	

36.27 0.35	-1.63 0.30	0.27	
***CMC* (γ) / mol·kg^{-1}**	**0.012**		
$C_{12}BetC_2Cl$ at *t* = 15 °C (below CMC)			
ρ_0 / (g·cm^{-3})	ρ_1 / (g·cm^{-3}·kg·mol^{-1})	ρ_2 / (g·cm^{-3}·kg^2·mol^{-2})	$\delta\rho$
0.9990970 ± 0.0000020	0.00755 ± 0.00043	-	0.000004
c_0 / (m·s^{-1})	c_1 / (m·s^{-1}·kg·mol^{-1})	c_2 / (m·s^{-1}·kg^2·mol^{-2})	δc
1466.351 ± 0.011	326.2 ± 2.7	-	0.03
a	*b*	$\delta\gamma$	
8.08 ± 2.66	-17.62 ± 0.99	0.98	
$C_{12}BetC_2Cl$ at *t* = 15 °C (above CMC)			
ρ_0 / (g·cm^{-3})	ρ_1 / (g·cm^{-3}·kg·mol^{-1})	ρ_2 / (g·cm^{-3}·kg^2·mol^{-2})	$\delta\rho$
0.9992530 ± 0.0000030	-0.00425 ± 0.00005	0.00211 ± 0.00015	0.000003
***CMC* (ρ) / mol·kg^{-1}**	**0.013**		
c_0 / (m·s^{-1})	c_1 / (m·s^{-1}·kg·mol^{-1})	c_2 / (m·s^{-1}·kg^2·mol^{-2})	δc
1469.852 ± 0.060	61.11 ± 0.38	-	0.09
***CMC* (*c*) / mol·kg^{-1}**	**0.013**		
a	*b*	$\delta\gamma$	
37.51 ± 0.32	-1.70 ± 0.29	0.29	
***CMC* (γ) / mol·kg^{-1}**	**0.014**		
$C_{12}BetC_2Cl$ at *t* = 25 °C (below CMC) (2)			
ρ_0 / (g·cm^{-3})	ρ_1 / (g·cm^{-3}·kg·mol^{-1})	ρ_2 / (g·cm^{-3}·kg^2·mol^{-2})	$\delta\rho$
0.9970540 ± 0.0000010	0.00420 ± 0.00017	-	0.000002
c_0 / (m·s^{-1})	c_1 / (m·s^{-1}·kg·mol^{-1})	c_2 / (m·s^{-1}·kg^2·mol^{-2})	δc
1496.666 ± 0.015	272.081 ± 3.6	-	0.03
a	*b*	$\delta\gamma$	
2.9 ± 3.3	-19.0 ± 1.2	1.3	
$C_{12}BetC_2Cl$ at *t* = 25 °C (above CMC) (2)			
ρ_0 / (g·cm^{-3})	ρ_1 / (g·cm^{-3}·kg·mol^{-1})	ρ_2 / (g·cm^{-3}·kg^2·mol^{-2})	$\delta\rho$
0.9971840 ± 0.0000030	-0.006597 ± 0.000046	0.002768 ± 0.000144	0.000003
***CMC* (ρ) / mol·kg^{-1}**	**0.012**		
c_0 / (m·s^{-1})	c_1 / (m·s^{-1}·kg·mol^{-1})	c_2 / (m·s^{-1}·kg^2·mol^{-2})	δc
1499.541 ± 0.042	40.11 ± 0.27	-	0.06
***CMC* (*c*) / mol·kg^{-1}**	**0.013**		
a	*b*	$\delta\gamma$	

36.07 ± 0.31	-1.67 ± 0.29	0.28	
***CMC* (*γ*) / mol·kg^{-1}**	**0.012**		
$C_{12}BetC_2Cl$ at *t* = 35 °C (below CMC)			
ρ_0 / (g·cm^{-3})	ρ_1 / (g·cm^{-3}·kg·mol^{-1})	ρ_2 / (g·cm^{-3}·kg^2·mol^{-2})	$\delta\rho$
0.994053 ± 0.0000010	0.00048 ± 0.00010	-	0.000001
c_0 / (m·s^{-1})	c_1 / (m·s^{-1}·kg·mol^{-1})	c_2 / (m·s^{-1}·kg^2·mol^{-2})	δc
1519.604 ± 0.070	240 ± 17	-	0.16
a	*b*	$\delta\gamma$	
6.82 ± 3.91	-16.88 ± 1.45	1.6	
$C_{12}BetC_2Cl$ at *t* = 35 °C (above CMC)			
ρ_0 / (g·cm^{-3})	ρ_1 / (g·cm^{-3}·kg·mol^{-1})	ρ_2 / (g·cm^{-3}·kg^2·mol^{-2})	$\delta\rho$
0.9941600 ± 0.0000030	-0.008479 ± 0.000045	0.003299 ± 0.000142	0.000003
***CMC* (*ρ*) / mol·kg^{-1}**	**0.012**		
c_0 / (m·s^{-1})	c_1 / (m·s^{-1}·kg·mol^{-1})	c_2 / (m·s^{-1}·kg^2·mol^{-2})	δc
1522.203 ± 0.035	19.58 ± 0.22	-	0.05
***CMC* (*c*) / mol·kg^{-1}**	**0.012**		
a	*b*	$\delta\gamma$	
35.60 ± 0.24	-1.55 ± 0.22	0.22	
***CMC* (*γ*) / mol·kg^{-1}**	**0.013**		
**$C_{12}BetC_2Cl$ at *t* = 45 °C (below CMC)**			
ρ_0 / (g·cm^{-3})	ρ_1 / (g·cm^{-3}·kg·mol^{-1})	ρ_2 / (g·cm^{-3}·kg^2·mol^{-2})	$\delta\rho$
0.9902380 ± 0.0000010	-0.001961 ± 0.000316	-	0.000003
c_0 / (m·s^{-1})	c_1 / (m·s^{-1}·kg·mol^{-1})	c_2 / (m·s^{-1}·kg^2·mol^{-2})	δc
1536.644 ± 0.006	185.0 ± 1.5	-	0.01
a	*b*	$\delta\gamma$	
8.1 ± 4.0	-15.9 ± 1.5	1.6	
$C_{12}BetC_2Cl$ at *t* = 45 °C (above CMC)			
ρ_0 / (g·cm^{-3})	ρ_1 / (g·cm^{-3}·kg·mol^{-1})	ρ_2 / (g·cm^{-3}·kg^2·mol^{-2})	$\delta\rho$
0.9903370 ± 0.0000030	-0.010018 ± 0.000049	0.00375 ±0.00016	0.000003
***CMC* (*ρ*) / mol·kg^{-1}**	**0.012**		
c_0 / (m·s^{-1})	c_1 / (m·s^{-1}·kg·mol^{-1})	c_2 / (m·s^{-1}·kg^2·mol^{-2})	δc
1538.913 ± 0.051	0.10 ± 0.32	-	0.08
***CMC* (*c*) / mol·kg^{-1}**	**0.012**		
a	*b*	$\delta\gamma$	
34.95 ± 0.25	-1.64 ± 0.23	0.22	

***CMC* (γ) / mol·kg^{-1}**	**0.013**

Table S4. Apparent molar volume, V_ϕ, adiabatic compressibility, κ_S, and apparent molar compressibility, $K_{S\phi}$ of *N*-alkyl betaine ethyl esters chlorides, C_nBetC_2Cl (for n = 6, 8, 10, 12 (1)) in aqueous solutions at 25 °C, and for C_{12}BetC_2Cl at temperatures t = (15 – 45) °C, with a step of 10 °C

m / (mol·kg^{-1})	$V_\phi \cdot 10^6$ / (m^3·mol^{-1})	$\delta V_\phi \cdot 10^6$ / (m^3·mol^{-1})	$\kappa_S \cdot 10^{10}$ / (Pa^{-1})	$K_{S\phi} \cdot 10^{13}$ / (m^3·mol^{-1}·Pa^{-1})
		C_6BetC_2Cl at t = 25 °C		
0.00020	231.96	1.1	4.47	-0.57
0.00058	235.07	0.3	4.47	-0.43
0.00095	235.58	0.2	4.47	-0.31
0.00232	237.79	0.07	4.47	-0.18
0.00488	237.88	0.03	4.47	-0.01
0.01103	238.37	0.01	4.46	-0.08
0.02046	238.36	0.01	4.45	-0.18
0.05421	238.47	0.003	4.41	-0.15
0.07805	238.37	0.002	4.38	-0.15
0.10062	238.24	0.002	4.35	-0.21
0.16739	238.07	0.001	4.28	-0.12
0.23096	237.86	0.001	4.22	-0.11
		C_8BetC_2Cl at t = 25 °C		
0.00010	260.16	2	4.47	-3.39
0.00020	258.29	2	4.47	-1.70
0.00050	261.66	0.5	4.47	-0.98
0.00098	266.86	0.2	4.47	-0.75
0.00194	269.01	0.07	4.47	-0.62
0.00504	268.89	0.03	4.46	-0.35
0.01006	269.45	0.01	4.46	-0.25
0.02005	269.79	0.01	4.44	-0.19
0.05038	269.65	0.002	4.40	-0.19
0.07673	269.55	0.002	4.37	-0.14
0.10098	269.36	0.001	4.34	-0.17
0.12353	269.50	0.001	4.31	-0.10
0.14823	269.68	0.001	4.29	-0.10
0.17219	269.43	0.001	4.26	-0.08
0.20934	269.87	0.001	4.23	-0.01
0.29177	269.90	0.0004	4.17	0.08
0.35362	270.65	0.0003	4.14	0.19
		C_{10}BetC_2Cl at t = 25 °C		
0.00008	295.46	2	4.48	-2.68

0.00022	299.77	0.4	4.48	-0.84
0.00053	301.28	0.2	4.48	-0.48
0.00094	301.32	0.1	4.48	-0.41
0.00199	301.48	0.04	4.47	-0.31
0.00528	301.56	0.02	4.47	-0.24
0.00991	301.54	0.01	4.46	-0.21
0.01964	301.62	0.004	4.45	-0.18
0.04802	301.80	0.002	4.41	-0.12
0.07519	303.60	0.001	4.39	0.15
0.10186	305.21	0.0004	4.38	0.39
0.14891	306.74	0.0002	4.37	0.62
0.18708	307.42	0.0001	4.36	0.72
0.31366	308.42	0.00004	4.33	0.88

$C_{12}BetC_2Cl$ at t = 25 °C (1)

0.00012	312.41	2.1	4.48	-5.05
0.00030	320.34	0.6	4.48	-1.91
0.00077	327.77	0.1	4.48	-0.80
0.00101	327.98	0.1	4.48	-0.68
0.00222	331.96	0.03	4.47	-0.33
0.00547	332.71	0.01	4.47	-0.26
0.01033	333.04	0.005	4.46	-0.19
0.01832	336.20	0.001	4.45	0.26
0.04753	340.75	0.001	4.45	0.92
0.07313	341.73	0.001	4.44	1.06
0.09783	342.14	0.001	4.44	1.12
0.17110	342.60	0.0004	4.42	1.20
0.25323	342.79	0.0003	4.41	1.23

$C_{12}BetC_2Cl$ at t = 15 °C

0.00007	225.93	15	4.65	-3.84
0.00022	286.59	2	4.65	-2.33
0.00045	302.95	0.8	4.65	-1.84
0.00100	318.31	0.2	4.65	-0.88
0.00241	324.18	0.05	4.65	-0.79
0.00483	326.71	0.02	4.64	-0.67
0.01012	328.01	0.01	4.63	-0.60
0.01992	332.06	0.003	4.62	-0.04
0.05141	337.27	0.0004	4.61	0.74
0.07442	338.23	0.0004	4.61	0.89
0.09959	338.74	0.0003	4.60	0.97
0.17494	339.38	0.0002	4.57	1.05
0.20120	339.46	0.0002	4.56	1.07
0.29168	339.64	0.0001	4.53	1.10

$C_{12}BetC_2Cl$ at t = 25 °C (2)

0.00007	267.71	10	4.48	-2.99
0.00022	305.21	2	4.48	-1.13
0.00045	314.65	0.5	4.48	-1.04

0.00100	325.93	0.1	4.48	-0.36
0.00241	329.42	0.03	4.47	-0.37
0.00483	331.32	0.01	4.47	-0.24
0.01012	332.16	0.006	4.46	-0.20
0.01992	336.54	0.001	4.45	0.35
0.05141	340.89	0.001	4.45	0.96
0.07442	341.70	0.001	4.44	1.07
0.09959	342.09	0.001	4.44	1.13
0.17494	342.64	0.0004	4.42	1.20
0.20120	342.70	0.0003	4.42	1.21
0.29168	342.84	0.0002	4.40	1.23
		$C_{12}BetC_2Cl$ at t = 35 °C		
0.00007	310.10	4.0	4.36	-1.95
0.00022	328.84	0.5	4.36	-0.43
0.00045	331.23	0.2	4.36	-0.38
0.00100	335.95	0.03	4.35	-0.09
0.00241	336.28	0.01	4.35	0.00
0.00483	336.70	0.005	4.35	0.09
0.01012	337.26	0.002	4.34	0.14
0.01992	340.77	0.002	4.34	0.62
0.05141	344.35	0.001	4.34	1.13
0.07442	345.02	0.001	4.34	1.22
0.09959	345.35	0.001	4.33	1.27
0.17494	345.76	0.0005	4.33	1.33
0.20120	345.82	0.0004	4.33	1.34
0.29168	345.92	0.0003	4.32	1.36
		$C_{12}BetC_2Cl$ at t = 45 °C		
0.00007	198.87	19	4.28	-1.30
0.00022	316.28	1	4.28	0.24
0.00045	314.40	0.6	4.28	0.12
0.00100	329.11	0.1	4.28	0.25
0.00241	338.42	0.008	4.27	0.35
0.00483	339.69	0.003	4.27	0.41
0.01012	340.38	0.002	4.27	0.42
0.01992	343.80	0.003	4.26	0.81
0.05141	347.34	0.002	4.27	1.27
0.07442	347.99	0.001	4.27	1.35
0.09959	348.31	0.001	4.27	1.40
0.17494	348.72	0.0006	4.27	1.46
0.20120	348.77	0.0005	4.27	1.47
0.29168	348.87	0.0003	4.27	1.48

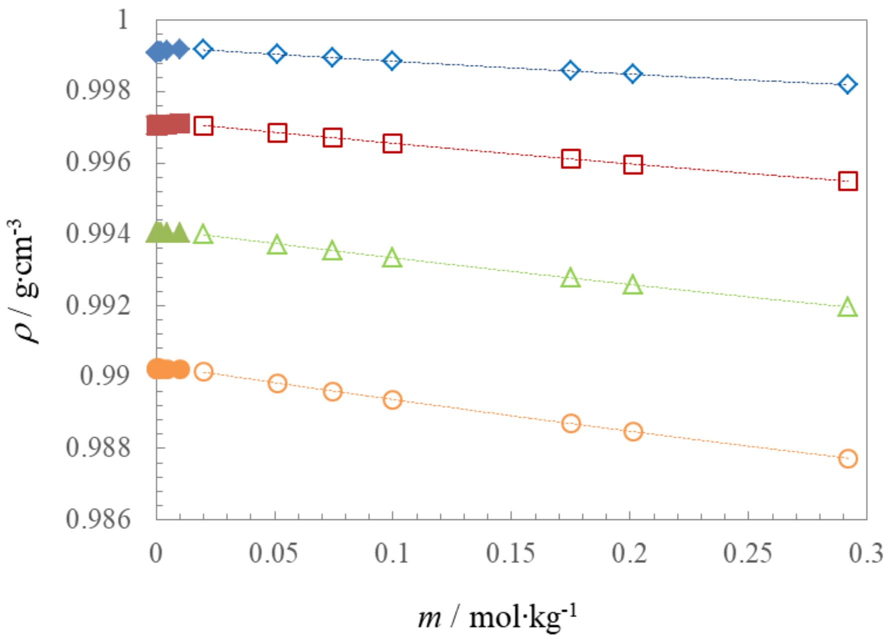

Figure S21. Density of aqueous solutions of $C_{12}BetC_2Cl$; experimental points: (♦, ◊) t = 15 °C, (■, □) t = 25 °C, (▲, △) t = 35 °C, (●, ○) t = 45 °C; filled points – before CMC, empty points – after CMC; lines – 1st or 2nd order polynomials (Table S3)

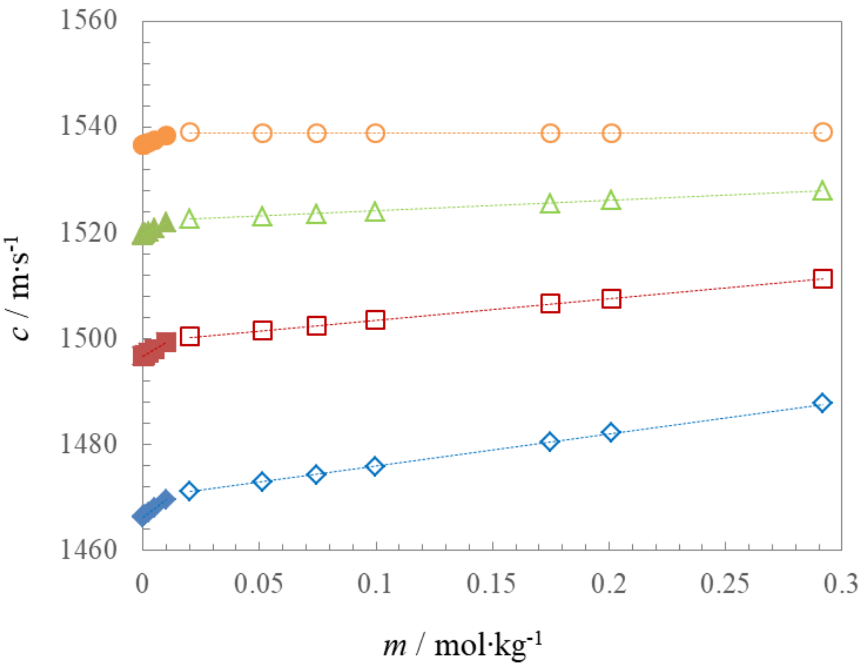

Figure S22. Speed of sound in aqueous solutions of $C_{12}BetC_2Cl$; experimental points: (♦, ◊) t = 15 °C, (■, □) t = 25 °C, (▲, △) t = 35 °C, (●, ○) t = 45 °C; filled points – before CMC, empty points – after CMC; lines – 1st order polynomials (Table S3)

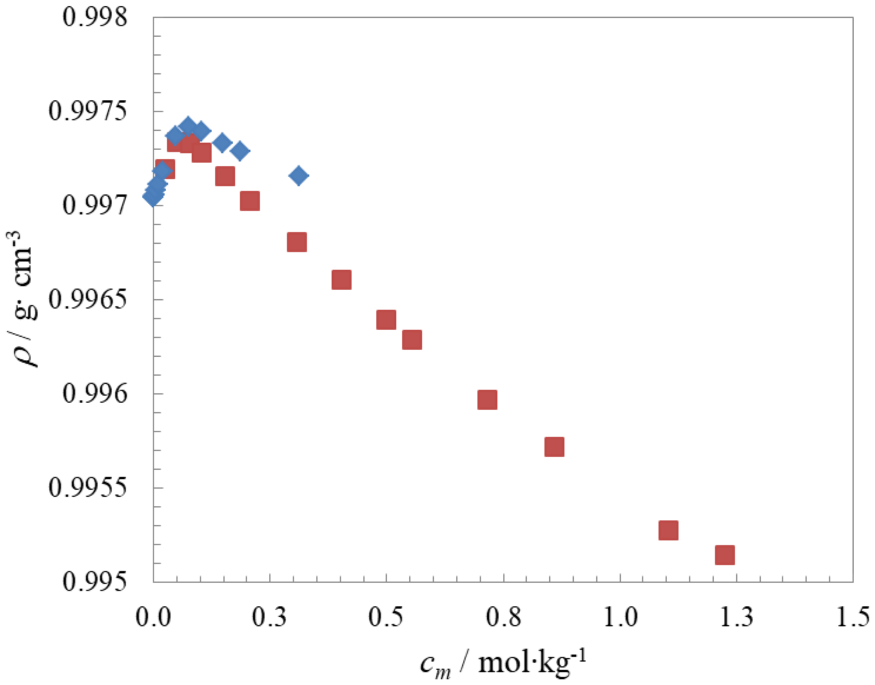

Figure S23. Comparison of density of aqueous solutions of $C_{10}BetC_2Cl$ at 25 °C; experimental points: (♦) this work, (■) from Ref. 22

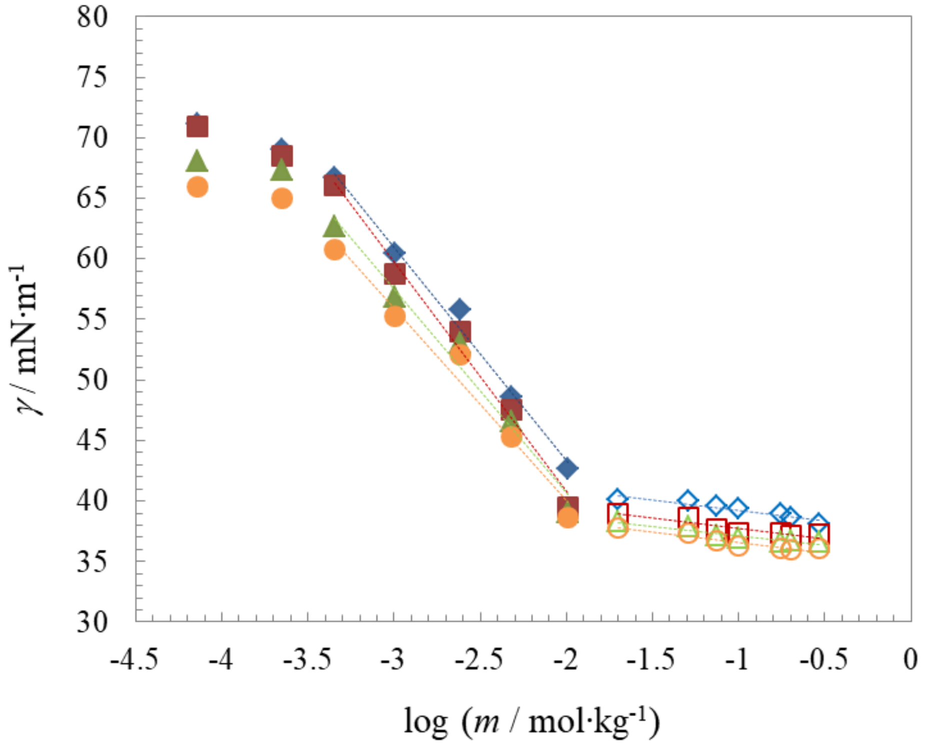

Figure S24. Surface tension of aqueous solutions of $C_{12}BetC_2Cl$; experimental points: (♦, ◊) t = 15 °C, (■, □) t = 25 °C, (▲, △) t = 35 °C, (●, ○) t = 45 °C; filled points – before CMC, empty points – after CMC; lines – 1st order polynomials (according Table S3)

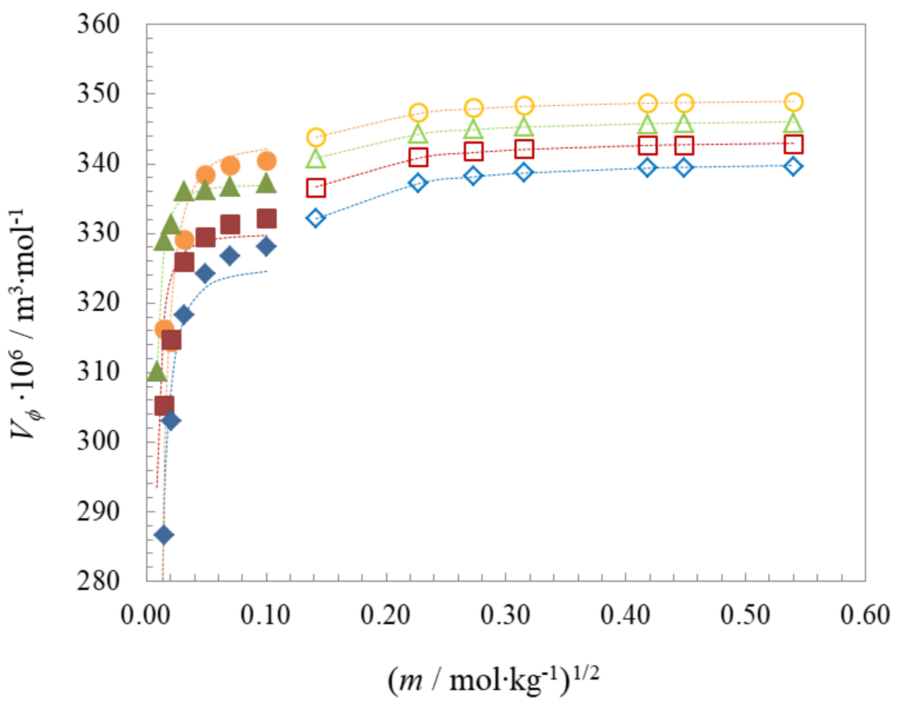

Figure S25. Apparent molar volume of $C_{12}BetC_2Cl$ in aqueous solutions; points: (♦, ◊) t = 15 °C, (■, □) t = 25 °C, (▲, ∆) t = 35 °C, (●, ○) t = 45 °C; filled points – before CMC, empty points – after CMC; lines – according Eq. (9)

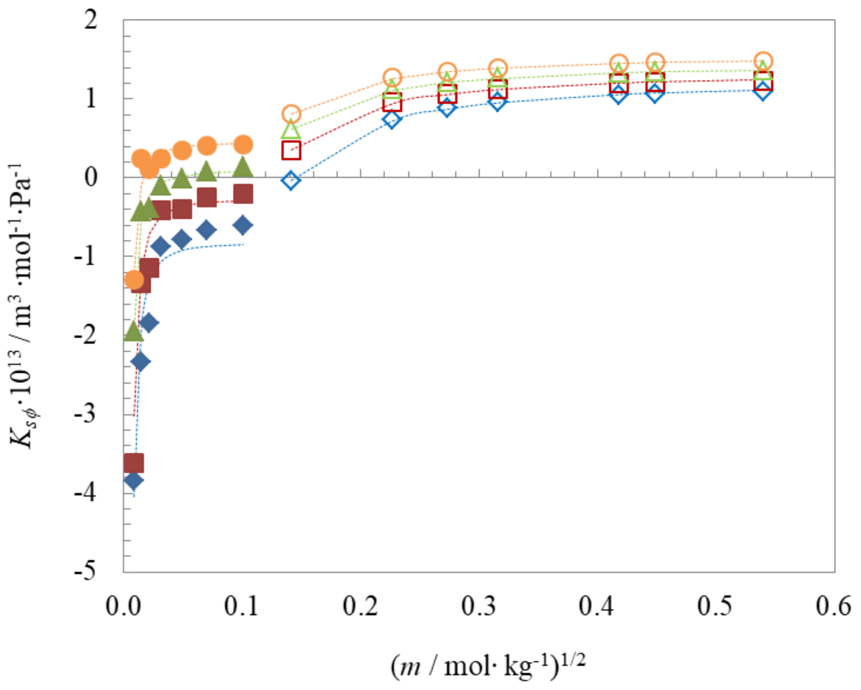

Figure S26. Apparent molar compressibility of $C_{12}BetC_2Cl$ in aqueous solutions; points: (♦, ◊) t = 15 °C, (■, □) t = 25 °C, (▲, ∆) t = 35 °C, (●, ○) t = 45 °C; filled points – before CMC, empty points – after CMC; lines – according Eq. (9)

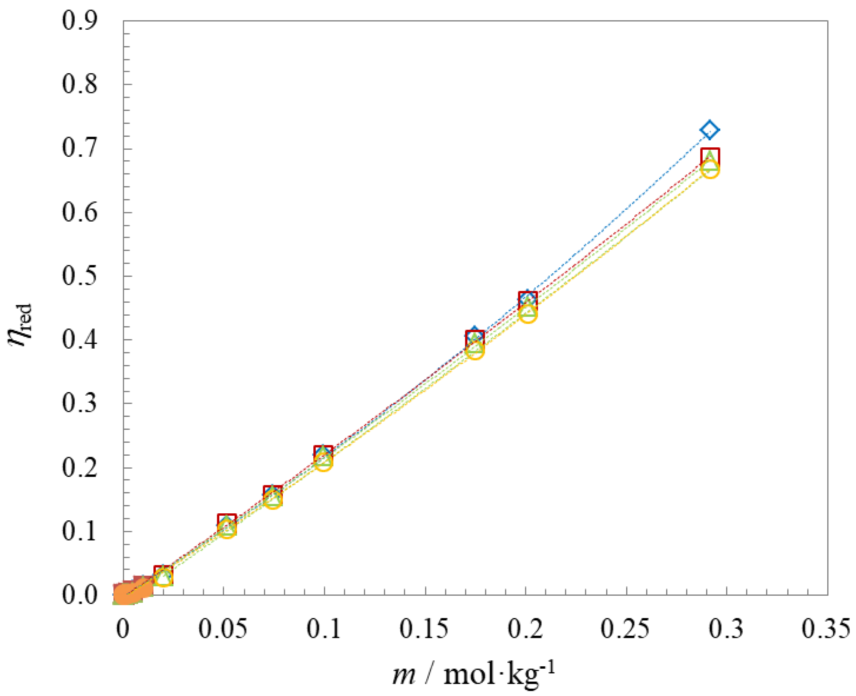

Figure S27. Reduced viscosity of aqueous solutions of $C_{12}BetC_2Cl$; points: (♦, ◊) t = 15 °C, (■, □) t = 25 °C, (▲, △) t = 35 °C, (●, ○) t = 45 °C; filled points – before CMC, empty points – after CMC; lines – according Eq. (10)
